# Effects of foot intensive rehabilitation (FIRE) on clinical outcomes for patients with chronic ankle instability: a randomized controlled trial protocol

**DOI:** 10.1186/s13102-023-00667-7

**Published:** 2023-04-09

**Authors:** Matthew C. Hoch, Jay Hertel, Phillip A. Gribble, Nicholas R. Heebner, Johanna M. Hoch, Kyle B. Kosik, Doug Long, Pinata H. Sessoms, Amy Silder, Danielle M. Torp, Katherine L. Thompson, John J. Fraser

**Affiliations:** 1 Sports Medicine Research Institute, University of Kentucky, 720 Sports Center Drive, Lexington, KY 40506 USA; 2 Sports Medicine and Chair, Department of Kinesiology, University of Virginia, 550 Brandon Avenue, Charlottesville, VA 22904-4407 USA; 3 Department of Physical Therapy, College of Health Sciences, University of Kentucky, 900 South Limestone, Lexington, KY 40536-0200 USA; 4 Warfighter Performance Department, Naval Health Research Center, 140 Sylvester Road, San Diego, CA 92106-3521 USA; 5 Dr. Bing Zhang Department of Statistics, University of Kentucky, 725 Rose Street, Lexington, KY 40536 USA; 6 Naval Health Research Center, 140 Sylvester Road, San Diego, CA 92106-3521 USA

**Keywords:** Ankle injury, Muscle, Plantar sensation, Therapeutics, Secondary prevention

## Abstract

**Background:**

Lateral ankle sprains account for a large proportion of musculoskeletal injuries among civilians and military service members, with up to 40% of patients developing chronic ankle instability (CAI). Although foot function is compromised in patients with CAI, these impairments are not routinely addressed by current standard of care (SOC) rehabilitation protocols, potentially limiting their effectiveness. The purpose of this randomized controlled trial is to determine if a Foot Intensive REhabilitation (FIRE) protocol is more effective compared to SOC rehabilitation for patients with CAI.

**Methods:**

This study will use a three-site, single-blind, randomized controlled trial design with data collected over four data collection points (baseline and post-intervention with 6-, 12-, and 24-month follow-ups) to assess variables related to recurrent injury, sensorimotor function, and self-reported function. A total of 150 CAI patients (50 per site) will be randomly assigned to one of two rehabilitation groups (FIRE or SOC). Rehabilitation will consist of a 6-week intervention composed of supervised and home exercises. Patients assigned to SOC will complete exercises focused on ankle strengthening, balance training, and range of motion, while patients assigned to FIRE will complete a modified SOC program along with additional exercises focused on intrinsic foot muscle activation, dynamic foot stability, and plantar cutaneous stimulation.

**Discussion:**

The overall goal of this trial is to compare the effectiveness of a FIRE program versus a SOC program on near- and long-term functional outcomes in patients with CAI. We hypothesize the FIRE program will reduce the occurrence of future ankle sprains and ankle giving way episodes while creating clinically relevant improvements in sensorimotor function and self-reported disability beyond the SOC program alone. This study will also provide longitudinal outcome findings for both FIRE and SOC for up to two years. Enhancing the current SOC for CAI will improve the ability of rehabilitation to reduce subsequent ankle injuries, diminish CAI-related impairments, and improve patient-oriented measures of health, which are critical for the immediate and long-term health of civilians and service members with this condition.

*Trial Registration* Clinicaltrials.gov Registry: NCT #NCT04493645 (7/29/20).

**Supplementary Information:**

The online version contains supplementary material available at 10.1186/s13102-023-00667-7.

## Background

In the US civilian population, lateral ankle sprains occur at a rate of 2 per 1000 person-years, which creates lifetime costs ranging from $9,196 to $11,925 per patient [1, 2]. The burden of ankle sprains is even higher in military personnel, with the incidence found to be up to 13 per 1000 person-years in officers and 29 per 1000 person-years in enlisted service members [3], representing 13% of all musculoskeletal injuries incurred by this population [4, 5]. The associated morbidity of lateral ankle sprains is compounded by the 40% of patients who subsequently develop chronic ankle instability (CAI), which is characterized by ongoing pain, ankle joint instability, repetitive injury recurrence, and persistent functional disability [6]. The symptoms and recurrence experienced by individuals with CAI are a consequence of persistent mechanical and neurophysiological impairments [7, 8], which contribute to early onset post-traumatic ankle joint osteoarthritis [9–12], deteriorations in physical activity, and declines in health-related quality of life that persist throughout the lifespan [13–17]. For individuals with CAI who seek medical care, management usually consists of palliative medication, basic rehabilitation exercises, and activity modification [18, 19]. Given the relative recalcitrance of CAI and the impact of persistent symptoms on joint and general health, it is clear that the standard of care (SOC) is inadequate for many patients [20].

Impaired joint motion, sensorimotor function, and balance are thought to contribute to repetitive joint trauma and short- and long-term self-reported disability in individuals with CAI [7, 8]. As a result, balance training, ankle strengthening, and range of motion exercises have become the core tenets of the standard of care for CAI rehabilitation [19, 21, 22]. While deficits in balance, ankle strength, and range of motion are regularly targeted during rehabilitation for patients with CAI, recent studies have indicated that many patients do not achieve clinically relevant improvements in sensorimotor function or health-related quality of life [23, 24]. Based on these findings and the complex neurophysiological nature of CAI, the current SOC may not address the full continuum of impairment and disability for patients with CAI. Therefore, there is a critical need to develop rehabilitation strategies that target unheeded impairments to improve the immediate and long-term outcomes for patients.

The foot provides critical somatosensory input, local stability to maintain a base of support, and is an integral component for force generation and attenuation during high energy activities. Preliminary research has identified intrinsic foot muscle (IFM) atrophy and activation deficits [25]; decreased hallux and lesser toe strength [26], and diminished plantar cutaneous sensitivity [27] in patients with CAI. These findings suggest that local foot stability and sensory input, critical for maintaining postural control, may be compromised. Despite these findings, somatosensory, motor, and mobility deficits in the foot are not routinely addressed in rehabilitation.

Interventions targeting IFM activation and plantar cutaneous sensation have demonstrated potential for improving sensorimotor function and health-related quality of life in patients with CAI [28–31]. Clinically relevant levels of activation have been achieved within IFMs using a series of foot core exercises, which focus on foot doming and isolated toe movements [32]. These exercises can improve dynamic balance, somatosensory and proprioceptive acuity, and reduce the severity of perceived instability in patients with CAI following small scale randomized controlled trials [29, 30]. Additionally, plantar massage interventions targeting somatosensory input from the foot have demonstrated the ability to improve single limb balance and health-related quality of life in CAI patients [28]. However, IFM exercises and plantar massage have only been studied in CAI patients in isolation and longitudinal outcomes following intervention have been limited. The additive effect of foot-related interventions to other evidenced-based interventions frequently employed in the SOC has not been examined [19]. Combining foot-related interventions with balance training, strengthening, and range of motion exercises may lead to greater improvements in sensorimotor function, health-related quality of life, and recurrent ankle injury rates.

Addressing sensorimotor function by correcting foot impairments and enhancing local foot stability could provide key additives to the current SOC rehabilitation protocol that could help to achieve more successful clinical outcomes in patients with CAI. Therefore, the overall objective of this randomized controlled trial is to examine the effects of a 6-week Foot Intensive REhabilitation intervention (FIRE) on ankle sprain re-injury and giving way rates, sensorimotor function, and self-reported disability in patients with CAI. Our central hypothesis is that by addressing the sensorimotor deficits at the foot we will reduce the occurrence of future ankle sprains and ankle giving way episodes, create clinically relevant improvements in sensorimotor function, and reduce self-reported disability beyond the SOC intervention alone*.* This study will be guided by the following specific aims:

- Specific Aim 1 Determine if a 6-week FIRE intervention decreases recurrent ankle sprain rates, frequency of ankle giving way episodes, and perceived symptom severity relative to a SOC intervention in patients with CAI.
- Specific Aim 2 Determine if FIRE improves sensorimotor function (static and dynamic balance, IFM activation, ankle/toe strength, somatosensation) relative to SOC in patients with CAI.
- Specific Aim 3: Determine if FIRE improves self-reported disability (foot and ankle function, sport-related disablement, injury-related fear) relative to the SOC in patients with CAI.

## Methods

### Summary and design

This clinical trial will employ a multisite, single-blinded parallel group randomized controlled trial design where patients will enroll at one of three sites: the University of Kentucky, the University of Virginia, or Naval Hospital Camp Pendleton (in partnership with the study teams at Naval Health Research Center). The framework of this design is to assess the superiority of the FIRE intervention in conjunction with SOC over SOC alone. This clinical trial was registered in the United States National Library of Medicine through ClinicalTrials.gov (NCT04493645). Table 1 details the key information pertaining to the registered trial. Ethical approval was granted by the University of Kentucky Institutional Review Board (#58,500), with reliance agreements and ethical approvals granted from the University of Virginia and the Naval Health Research Center in compliance with the single IRB protocol. This protocol has also been reviewed and approved by the Human Research Protection Offices of the U.S. Army Medical Research and Development Command Office of Research Protections and the US Marine Corps. Informed consent will be obtained in writing from all patients prior to enrollment. This work was supported by the Congressionally Directed Medical Research Programs (820 Chandler St; Fort Detrick MD 21,702–5014; help@eBRAP.org; 301-682-5507), grant W81XWH-20-2-0035. Outside of the human research protection review, the funding sponsor does not have direct role in directing study design; collection, management, analysis, and interpretation of data; writing of the report; or the decision to submit the report for publication. The CONSORT Statement for Randomized Trials of Nonpharmacologic Treatments [33], the template for intervention description and replication (TIDieR) [34], and Standard Protocol Items: Recommendations for Interventional Trials (SPIRIT) 2013 [35] were used to guide reporting.

**Table 1:** Trial registration data

Data category	Information
Primary registry and trial identifying number	ClinicalTrials.gov NCT04493645
Date of registration in primary registry	30 July 2020
Secondary identifying numbers	58,500; CDMRP-OR190060 (Other Grant/Funding Number: Department of Defense)
Source(s) of monetary or material support	Congressionally Directed Medical Research Programs
Primary sponsor	Congressionally Directed Medical Research Programs
Secondary sponsor(s)	NA
Contact for public queries	Douglas Long, MS, 859–323-5438, delong2@uky.edu; Matthew Hoch, PhD, ATC, 859–323-9850, matt.hoch@uky.edu
Contact for scientific queries	Matthew Hoch, PhD, ATC, Sports Medicine Research Institute, University of Kentucky
Public title	Ankle Instability Using Foot Intensive Rehabilitation
Scientific title	Optimizing Clinical Outcomes for Patients with Chronic Ankle Instability Using Foot Intensive Rehabilitation
Countries of recruitment	United States
Health condition(s) or problem(s) studied	Ankle Injuries; Ankle Sprains
Intervention(s)	Active comparator: Foot Intensive Rehabilitation (FIRE) and Standard of Care Rehabilitation (SOC). 6 weeks of FIRE will be given along with elements of SOC. Each patient will be expected to complete 2 supervised sessions and 3 unsupervised at home sessions per week
	Control comparator: Standard of Care Rehabilitation. 6 weeks of SOC rehabilitation will be given designed to restore ankle joint range of motion, strength, postural control, and functional movement. Each patient will be expected to complete 2 supervised sessions and 3 unsupervised at home sessions per week
Key inclusion and exclusion criteria	Ages eligible for study: 18 to 44 years; Sexes eligible for study: both; Accepts healthy volunteers: no
	Inclusion criteria: Aged 18–44; Previous history of at least 1 ankle sprain and at least 2 episodes of "giving way" in the past 3 months; Patients must answer "yes" to at least 5 questions on the Ankle Instability Instrument; Score of 11 or higher on the Identification of Functional Ankle Instability (IdFAI); Confirmed clinical presentation of CAI by a PT, AT, or MD
	Exclusion criteria: Sustained an ankle sprain in the previous four weeks or lower extremity neuromusculoskeletal injury other than to the ankle in the last 12 months; History of surgery to the lower extremity; Sustained a lower extremity fracture; History of neurological disease, vestibular or visual disturbance or any other pathology that would impair their sensorimotor performance; Current participation in a formal ankle joint rehabilitation program; Sustained a concussion in the last 12 months; Exhibit clinical examination characteristics of foot and ankle function which are consistent with conditions other than CAI (i.e. fracture, deformity)
Study type	Allocation: Randomized; Intervention Model: Parallel Assignment; Intervention Model Description:The investigators will compare clinical and innovative outcome measures collected at five time points between cohorts of patients with CAI that receive a standard of care (SOC) rehabilitation program compared to an innovative foot intensive rehabilitation (FIRE) program to determine if FIRE can further reduce the rate of re-injury, improve sensorimotor function, and reduce self-reported disability during the 24 months following the intervention.; Masking: Single (Outcomes Assessor); Masking Description: The investigators collecting the outcomes will be blinded to group allocation. Separate investigators will be used for intervention delivery and outcomes assessment. Primary Purpose: Treatment
Date of first enrolment	October 2021
Target sample size	150
Recruitment status	Recruiting
Primary outcome(s)	Number of recurrent ankle sprains [Time Frame: 24 months]: The ability of FIRE to attenuate the occurrence of ankle sprains compared to SOC rehabilitation will be determined through self-report. An ankle sprain will be operationally defined as an incident in which the rearfoot was inverted or supinated and resulted in a combination of swelling, pain, and time lost or modification of normal function for at least one day Frequency of episodes of the ankle giving way [Time Frame: 24 months]: The ability of FIRE to attenuate the number of episodes of the ankle giving way compared to SOC rehabilitation will be determined through self-reported occurrences per week in the past month. Episodes of giving way will be operationally defined for the patient as an incident in which the rearfoot suddenly rolled, felt weak, or lost stability; however, the individual did not sustain an ankle sprain and was able to continue with normal function Severity of chronic ankle instability related symptoms [Time Frame: 24 months]: The ability of FIRE to attenuate the severity of related symptoms compared to SOC rehabilitation will be determined through the Cumberland Ankle Instability Tool. The Cumberland Ankle Instability Tool is a 9-item instrument used to identify self-reported impairments associated with CAI. This instrument is scored on a 0–30 scale, where lower scores represent greater severity of CAI related symptoms
Key secondary outcomes	Postural Control [Time Frame: 12 months]: The ability of FIRE to improve static and dynamic postural control compared to SOC rehabilitation will be determined. Multiple measurements will be made including: Single-limb stance on each limb with eyes open and eyes closed with use of a force plate for center of pressure measurements, Star Excursion Balance Test, forward jump single limb landing stabilization task. All measurements will be monitored while the patient wears an inertial sensor placed on the lumbar spine Ankle/Toe Strength [Time Frame: 12 months]: The ability of FIRE to improve strength compared to SOC rehabilitation will be determined. Strength of the muscles surrounding the ankle and the toes will be assessed through a series of assessments with a digital handheld dynamometer Intrinsic Foot Muscle Activation [Time Frame: 12 months]: The ability of FIRE to improve foot muscle activation compared to SOC rehabilitation will be determined. Abductor hallucis, flexor digitorum brevis, quadratus plantae, and flexor hallucis brevis functional activity ratios will be captured using diagnostic ultrasound with a 12–4 MHz linear array transducer probe and measured using Image J software Plantar Cutaneous Sensation [Time Frame: 12 months]: The ability of FIRE to improve plantar cutaneous sensation compared to SOC rehabilitation will be determined. Plantar cutaneous sensation will be tested using a 20-piece Semmes–Weinstein Monofilament kit which has monofilaments ranging from to 0.008 g of force (1.65 level) to 300 g of force (6.65 level). Light touch detection thresholds will be assessed on the plantar surface at the 1st metatarsal head

### Patients

One hundred and fifty men and women (*n* = 50 at each site) will be recruited from the campuses and surrounding communities associated with the University of Kentucky, University of Virginia, and Naval Hospital Camp Pendleton through posted flyers, social media postings, and word of mouth at local sports medicine clinics. The university-based sites are sports medicine laboratories located in suburban academic settings in Lexington, KY and Charlottesville, VA. The military site is an outpatient sports medicine clinic located at Marine Corps Base Camp Pendleton that provides specialized primary and referred care to military beneficiaries consisting primarily of US Marines and Navy Sailors. At all three sites, potential patients will be pre-screened by telephone or in-person by a local member of the research team using a checklist containing components of the inclusion and exclusion criteria. Patients who appear eligible based on prescreening and have continued interest in participating in the study will be scheduled to meet with a member of the research team who trained to perform the enrollment and informed consent process.

The procedures for assessing eligibility were derived and aligned with the guidelines for selecting CAI patients from the International Ankle Consortium [36]. To be included, patients must be a male or female adult, aged 18–44 years with a history of ≥ 1 ankle sprain. Patients must also report ≥ 2 episodes of “giving way” in the past 3 months. An ankle sprain will be defined as an injury in which the rearfoot was inverted or supinated and resulted in a combination of swelling, pain, and time lost or modification of normal function for at least one day [37]. Episodes of giving way will be described as an incident in which the rearfoot suddenly rolled, felt weak, or lost stability; however, the individual did not sustain an ankle sprain and will have been able to continue with normal function after the incident [37]. In addition, patients must answer “yes” to ≥ 5 questions on the Ankle Instability Instrument and ≥ 11 on the Identification of Functional Ankle Instability [38]. In cases of bilateral CAI, the limb with the higher Identification of Functional Ankle Instability score will be identified as the involved limb for intervention.

Patients will be excluded if their involved limb sustained an ankle sprain within four weeks, lower extremity injury within twelve months, history of lower extremity surgery or fracture, or concussion within 12 months, conditions other than ankle sprain that affect balance or cutaneous sensation, or they are receiving ankle rehabilitation at the time of screening. The investigator at each site completing the patient enrollment will also perform a basic clinical examination of the ankle that will include tests of ankle ligamentous laxity and joint restriction, foot and ankle fracture, point tenderness, and ankle–foot deformity. The investigators performing these procedures will have professional training in orthopaedic evaluation and will determine if the clinical presentation of the patient is consistent with CAI. If a patient exhibits signs of ankle–foot conditions that are not consistent with CAI, they will be excluded from participation.

### Procedures

**Fig. 1:**
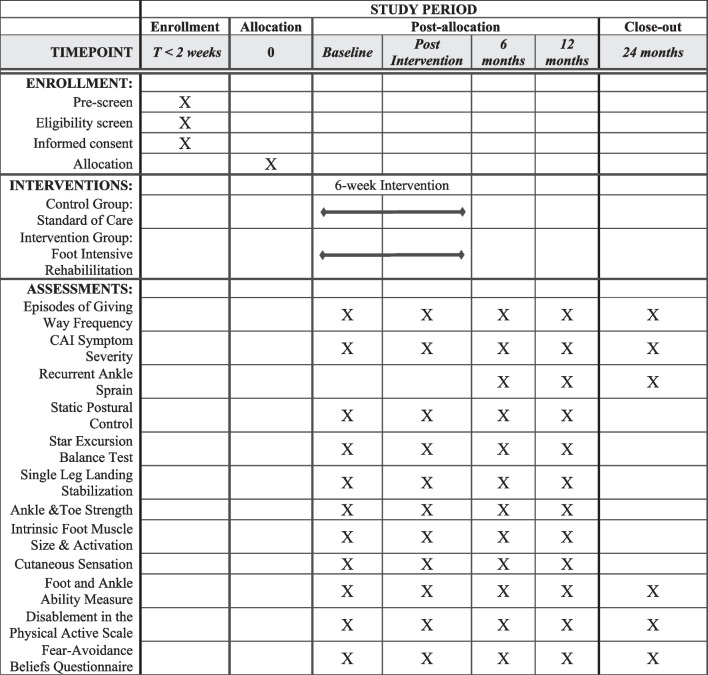
Schedule of enrolment, interventions, and assessments

The SPIRIT flow diagram detailing study procedures can be found in Fig. 1. Once a patient provides written consent and is deemed eligible to be enrolled in the study, they will be assigned a patient identification number and randomized to either the FIRE or SOC group. A randomization schedule will be prepared by an investigator (KLT) not involved in data collection or intervention delivery using statistical software (SAS v9.4 PROC PLAN). Within each site, 50 patients will be randomly assigned to one of two groups (FIRE or SOC group). To ensure balance in groups over time, randomization will be completed in sequential sets of 10 subjects (5 FIRE and 5 SOC) within each site. Randomization plans include an additional 30 subjects to account for anticipated attrition during the study. Once completed, the randomization plan will be provided to the study coordinator at each site. The investigators will be blinded to group allocation by concealing assignments in sealed opaque envelopes. The treating rehabilitation specialist will retrieve the group assignment from the envelope following collection of baseline data.

Data collection will occur at five different time points (baseline, post-intervention, 6-months, 12-months, and 24-months) for patients in both groups. Baseline testing will be completed after enrollment and prior to starting the assigned intervention. Patients will begin the intervention within one week of completing baseline testing. Post-intervention testing will occur within one week of completing the assigned rehabilitation program. Follow-up measures will be repeated longitudinally at approximately 6, 12, and 24 months after baseline testing (Fig. 1). A description of the specific outcomes associated with each of these aims is presented below. If any patients are unable to complete the follow-up sessions in person, the outcomes assessments for Aims 1 and 3 will be collected electronically to reduce attrition.

### Interventions

Patients in both the FIRE and SOC groups will complete two supervised and three unsupervised sessions during each week of the 6-week intervention, for a total of 12 supervised rehabilitation sessions and 18 unsupervised rehabilitation sessions. During the supervised sessions, patients will work directly with a credentialed rehabilitation specialist (physical therapist or athletic trainer) who was trained on the intervention procedures and demonstrated proficiency during prerecruitment calibration. The treating clinicians will schedule rehabilitation sessions with patients and record the date, duration of session, and exercises completed during each supervised session. Additionally, the treating clinician will record any reports of patient soreness, discomfort, or other symptoms and adjust the intervention consistent with the tenets of evidence-based practice. Patients will be instructed on how to complete the unsupervised exercises after the initial supervised session and will demonstrate the home exercises to the treating clinician before leaving the clinic to help ensure full understanding. To track compliance with unsupervised sessions, patients will be provided an exercise program and log to record the number of sessions, sets, and repetitions of exercises completed. Patients will be asked to demonstrate the exercises performed at home to assess recall and technique during each subsequent supervised session. Patients with reported non-compliance, limited recall, or reported displeasure with performance of the assigned exercises will be retrained and encouraged to continue with the allocated interventions. The treatment course will be modified (and annotated) as required based on the needs of the patient, treatment response, and patient preference. The home and supervised exercise library for both the SOC and FIRE programs have been included as supplements.

### Standard of care rehabilitation program

Details of the SOC exercise program are provided in the supplemental material (Standard of Care Supervised and Home Exercise Intervention Protocols). The supervised portion of the SOC will contain previously established balance training exercises, progressive 4-way (inversion, eversion, dorsiflexion, plantarflexion) ankle strengthening program using resistance bands [39–41], hip strengthening program using resistance bands and rotational movements, talocrural joint mobilization, and triceps surae stretching [23]. This combination of treatment exercises represented the most common rehabilitation techniques for CAI and was developed based on previous clinical trials [23, 41–43]. These exercises were recommended in a recently published clinical practice guideline for assessment and treatment of ankle sprains and instability [44].

The evidence-based dynamic balance training exercises include: (1) single-limb static balance, (2) single-limb hops to stabilization, (3) hop to stabilization and reach, and (4) unanticipated hop to stabilization. Static balance exercises will include single-limb stance with eyes opened and closed on firm and foam surfaces. Starting points will be individually determined, but the performance-based progression for each exercise will follow a previously established protocol [42]. Strengthening exercises for dorsiflexion, plantar flexion, inversion, and eversion of the ankle and flexion, extension, adduction, and abduction of the hip will be completed using resistance bands [40, 41, 43]. Patients will use a heavy band during the first two weeks, an extra heavy band during the middle two weeks, and a special heavy band for the last two weeks of the intervention [41]. The number of sets and repetitions completed during each treatment session will be progressed based on a previous protocol [41]. Finally, to address range of motion, patients will receive talocrural joint mobilization, triceps surae stretching, and wobble-board training. Joint mobilizations will consist of two, 2-min sets of Maitland Grade III anterior-to-posterior talocrural joint mobilizations with 1-min of rest between sets [45]. The triceps surae stretching will consist of three sets of 30-s of stretching with the knee in full extension as well as three sets with slight knee flexion to target the gastrocnemius and soleus muscles [46]. Range of motion will also be targeted using a progressive wobble board protocol, which will progress from sitting, double limb stance, and single limb stance. The unsupervised sessions for the SOC protocol will consist of components extracted from the supervised rehabilitation session including single-limb balance, resistance band, and triceps surae stretching exercises.

### Foot intensive rehabilitation program

Details of the FIRE intervention can be found in the Additional file 1 (FIRE Supervised and Home Exercise Intervention Protocol). The FIRE intervention will include the progressive balance training, ankle and hip strengthening, and range of motion exercises from the SOC intervention; however, several exercises will be modified and added that concentrate on foot muscle activation, plantar cutaneous somatosensory feedback, and the integration of foot stability during movement. Plantar massage will consist of two, 1-min plantar massages with a 1-min rest between sets. This massage will be a combination of effleurage and petrissage techniques to the entire plantar aspect of the foot with the patient supine [28]. Four previously established exercises will target the IFMs including the short-foot, toe-spread-out, hallux extension, and lesser-toe extension [31, 32, 47]. In the first treatment session, patients will start each exercise in a seated position. Progression to double-limb stance and single-limb stance will occur when an exercise is done correctly for an entire session without compensation. A series of exercises will also target the extrinsic foot muscles involved in foot posture including resistive band supination and pronation, step ups with active supination or pronation and bilateral heel raises with a ball squeeze between the heels [48, 49]. The balance training exercises described for the SOC intervention will be completed during the FIRE intervention; however, the FIRE group will be instructed by the supervising interventionist to emphasize IFM activation during static balance exercises and after landing during dynamic balance exercises. The unsupervised sessions for the FIRE intervention will consist of single-limb balance, triceps surae stretching, supination and pronation resistance band, and intrinsic foot muscle exercises. Additionally, plantar massage will be self-administered by rolling the plantar surface of the foot on a textured massage ball on the ground [50].

### Outcome measures

Separate members of the study team who are trained in the assessments, demonstrated proficiency during pre-collection calibration, and blinded to group assignment will collect all outcomes. Blinding of the assessors will be maintained for the duration of data collection to avoid bias throughout the study timeline. The schedule for the collection of each outcome measure can be found in Fig. 1. Details of the outcome measures are included below.

### Primary outcomes

#### Recurrent ankle sprain and episodes of giving way

The number of recurrent ankle sprains since the previous testing session and the average number of ankle giving way episodes per week over the past month will be assessed through self-reporting. An ankle sprain will be operationally defined as an incident in which the rearfoot was inverted or supinated and resulted in a combination of swelling, pain, and time lost or modification of normal function for at least one day [37]. Episodes of giving way will be operationally defined as an incident in which the rearfoot suddenly rolled, felt weak, or lost stability; however, the individual will not have sustained an ankle sprain and was able to continue with normal function.

### Cumberland ankle instability tool

The Cumberland Ankle Instability Tool is a 9-item instrument used to identify self-reported impairments associated with CAI [51]. This instrument is scored on a 0–30 scale, where lower scores represent greater severity of CAI related symptoms [51]. The questions encompass various impairment areas associated with CAI including ankle pain, frequency of feeling unstable during activity, ability to control moments of instability, and perceived recovery time from episodes of instability. In development, this instrument demonstrated acceptable construct validity, internal reliability, test–retest reliability (ICC = 0.96), and could effectively discriminate between patients with and without CAI [51].

### Secondary outcomes

#### Static balance

Static postural control will be assessed with the Accusway Plus force plate (AMTI; Watertown, MA). Force and moment signals will be sampled at 100 Hz and converted to center of pressure estimates through Balance Clinic Software (AMTI, Watertown, MA, USA). Center of pressure data will be subsequently low-pass filtered at 5 Hz (Butterworth, 4th order, zero lag) through the Balance Clinic Software. Patients will perform one practice trial and three analysis trials of single-limb stance on each limb with eyes open and eyes closed for 20 s, for a total of 12 analysis trials. Patients will be instructed to stand with their arms folded across their chest, the uninvolved limb lifted off the force plate, positioned at approximately 45° of knee flexion, and the hip flexed to approximately 30°. If the patient touches down with the suspended limb, opens their eyes during eyes closed testing, or is unable to maintain the standing posture for the 10 s duration, the trial will be discarded and repeated. Center of pressure data will be separated into anterior–posterior (AP) and medial–lateral (ML) components and analyzed as AP and ML velocity, area 95% eclipse, and time-to-boundary (TTB) using a custom MATLAB code (The Mathworks, Natick, MA, USA) [42].

### Star excursion balance test

The Star Excursion Balance Test will be used as a clinical assessment to measure dynamic postural control. To complete this test, patients will place hands on hips, balance on the involved limb and reach with the uninvolved limb in the anterior, posteromedial, and posterolateral directions as far as possible. Trials will be discarded and repeated if the patient fails to maintain balance, lifts the heel, removes hands from hips, places weight on the reaching limb during toe touch, or fails to return to the starting position. Patients will complete four practice and three analysis trials in each direction on both limbs [52]. Collection trials will be averaged and normalized to leg length. Longer reach distances will represent greater dynamic postural control.

### Hop-to-stabilization

Dynamic postural control will also be measured using a forward jump hop-to-stabilization task [53, 54]. To complete this task, patients will initiate a double-leg forward jump and land on a single-leg. Patients were instructed to jump over a 30 cm hurdle placed at half the distance from the starting position to the target landing area (60 cm × 90 cm). The minimum jump distance (starting line to target landing area threshold) will be normalized to 40% of the person’s height. Patients will be instructed to land, obtain their balance, place their hands on the hips, and remain as still as possible for five seconds. Three successful trials will be recorded. Trials will be repeated if they do not land completely in the target area, touch down with the other foot, or move the stance leg after landing. Prior to beginning the task, an inertial measurement unit (Xsens DOT, V2.0.0, Xsens Technologies B.V., The Netherlands) will be secured to the low back (L4/5). Tri-axial acceleration data from this sensor will be sampled at 60 Hz. Using a custom MATLAB code (The Mathworks, Natick, MA, USA), dynamic postural stability index values will be estimated as the root mean square for accelerometer data in each orthogonal direction (AP, ML, Vertical) and resultant magnitude.

### Ankle and toe strength

Muscle strength will be assessed with the MicroFET2 digital handheld dynamometer (Hoggan Scientific LLC, Salt Lake City, UT, USA) using previously described methods [55]. Briefly, ankle dorsiflexion will be assessed in the longsit position with the dynamometer placed over the dorsal metatarsal heads. Ankle inversion and eversion will be assessed in the longsit position with the dynamometer placed on the medial and lateral forefoot, respectively. Ankle plantarflexion will be assessed with the patient laying prone and dynamometer placed on the plantar metatarsal heads. Lastly, the hallux and lesser toe flexion will be assessed with the patient’s forefoot suspended off the table with their heel flat and the dynamometer placed under the hallux or lesser toes. Strength measures will be based on a single trial of a “make test” and reported in Newtons (N). In the case of an invalid trial (due to equipment difficulty, deviation from test position, or compensatory motion), the patient will be allowed rest prior to retesting to mitigate effects from fatigue.

### Intrinsic foot muscle activation

Abductor hallucis, flexor digitorum brevis, quadratus plantae, and flexor hallucis brevis thickness and functional activation ratios will be captured using ultrasound imaging and measured using WebPlotDigitizer software version 4.6 (Ankit Rohatgi, https://automeris.io/WebPlotDigitizer, Pacifica, CA, USA). The patient will be positioned supine with the plantar aspect of the foot exposed. The shank will be secured to a bolster to standardize patient positioning. The assessor will ensure both the forefoot and rearfoot are neutrally positioned in both sagittal and frontal planes during scanning. The ultrasound transducer placement will be standardized based on a previously described protocol [56]. The gain will be adjusted to ensure fascial borders of the IFM are identifiable. Initial measurements will be taken at rest with no contraction of the IFM. These measures will be followed by open kinetic chain isometric contractions of hallux abduction for the abductor hallucis (resistance applied at medial distal phalanx), lesser toe flexion for the flexor digitorum brevis and quadratus plantae (resistance applied at distal pads of toes 2–5), and hallux flexion for the flexor hallucis brevis (resistance applied at distal pad of great toe). Thickness measurements will be taken at rest and while activated based on previously described procedures [56]. The functional activity ratio will be calculated to measure IFM activation. An activation ratio of > 1.00 will indicate an increase in muscle size and < 1.00 will indicate a decrease in muscle size. This protocol has previously documented excellent reliability for these measures (ICC ≥ 0.87) [56].

### Plantar cutaneous sensation

Plantar cutaneous sensation will be tested using a 20-piece Semmes–Weinstein Monofilament kit (Touch-Test Sensory Evaluator; North Coast Medical, Gilroy, CA, USA) which has monofilaments ranging from to 0.008 g (1.65 level) to 300 g (6.65 level). Light touch detection thresholds will be assessed on the plantar surface at the 1st metatarsal head. Patients will lay prone with noise reducing headphones and asked to respond “yes” when they perceive a monofilament. Monofilaments will be applied perpendicular to the skin with the fiber bent to a “C” shape. Detection thresholds will be identified using a previously established 4–2-1 stepping algorithm method [57]. The detection threshold will be the lightest weight monofilament perceived by the subject. This protocol has demonstrated acceptable intrarater (ICC = 0.61–0.85) and interrater reliability (ICC = 0.62–0.92) [57].

### Foot and ankle ability measure

The Foot and Ankle Ability Measure is a region-specific patient-reported outcome used to assess functionality in patients with leg, ankle, or foot pathology with questions pertaining to the patient’s function level while performing activities of daily living and sport-related activities. The activities of daily living and sport subscales contain 21 items and 8 items respectively, and each scale is scored independently [58]. Each item is scored on a 5-point Likert scale where 0 indicates “*no problem*” and 4 indicates *“unable to do*”. The final score is often reported as a percentage of the total score, where lower scores indicate decreased self-reported function. The Foot and Ankle Ability Measure is reliable, valid and responsive in quantifying progress of patients with a wide range of foot and ankle pathologies [58]. Test–retest reliability is acceptable for both the activities of daily living (ICC = 0.89) and the sport (ICC = 0.87) subscales [58]. The minimal detectable change for the activities of daily living scale and sport subscales are ± 5.7 and ± 12.3 points, respectively [58].

### Modified disability in the physically active scale

The Disablement in the Physically Active Scale (DPA) [59] is a generic 16-item patient-reported outcome instrument that assesses physical and psychosocial status for physically active adults. A modified version of this instrument was restructured to separate the items into a psychosocial (Mental Composite Score) and a physical component (Physical Composite Score) [60]. Each item is scored on a 4-point Likert scale with 0 indicating *no problem* and 4 indicating *severely affected*. Although the items are the same as the original, the two components are scored independently. Thus, the Physical and Mental Composite Scores range from 0 to 48 and 0 to 16, respectively, with higher scores indicating greater disability. Adequate internal consistency was demonstrated for both the Physical (*α* = 0.941) and Mental Composite Scores (*α* = 0.878). The minimal detectable change scores of the Physical and Mental Composite Scores in individuals with CAI is 7 and 3 points, respectively [23].

### Fear avoidance beliefs questionnaire

The Fear-Avoidance Beliefs Questionnaire-Physical Activity subscale is designed to assess fear avoidance beliefs associated with physical activity in patients who are injured or who have a history of injury. This is a 5-item instrument scored on a 7-point scale with responses ranging from ‘*completely disagree*’ to ‘*completely agree*’. Scores range from 0 to 24 with a higher score representing increased fear avoidance [61]. This instrument has previously demonstrated sound clinometric properties including strong internal consistency and test–retest reliability (*a* = 0.77–0.96) with a minimal detectable change of 4 points in those with CAI [62, 63].

### Power analysis

The primary comparisons for all aims are the comparisons between FIRE and SOC groups at 6 months for the CAIT (Aim 1), posteromedial reach direction of the SEBT (Aim 2), and the Foot and Ankle Ability Measure Sport (Aim 3). A two-sample t-test comparing the change score between the FIRE and SOC groups will have at least 95% power to detect an effect size of 0.6 between the group means when the sample size is 150 (75 per group), assuming a two-sided significance level of 0.05. In the case that attrition reaches 40%, the sample size will still allow for 80% power to detect the same effect.

### Statistical plan and data analysis

Continuous variables will be summarized with descriptive statistics and categorical variables will be summarized with counts and percentages. Change scores and percent change scores will be calculated from baseline for follow-ups at post-intervention, 6-, 12-, and 24-months; the primary outcome for all aims is the 6-month change score. Simple comparisons between groups will be performed using two-sample t-tests for continuous variables and chi-square tests of independence for categorical variables. Effect sizes will be interpreted as weak (≤ 0.39), moderate (0.40–0.69), or strong (≥ 0.70). A two-sided significance level of 0.05 will be used for all statistical tests. All analyses will be completed with SAS v9.4 (SAS Institute, Cary, NC, USA).

Although groups will be randomly assigned, potential covariates will be examined with bivariate analyses, and comparisons requiring covariate adjustment will use regression modeling (e.g. ANCOVA, logistic regression); unadjusted and adjusted estimates will be presented with 95% confidence intervals. Potential covariates include: baseline outcome values, demographic variables (e.g., sex, age, height, weight, data collection site), prognostic indicators (e.g., number of previous ankle sprains, frequency of episodes of giving way, Identification of Functional Ankle Instability score), and intervention compliance (% sessions completed).

To examine the trajectory over time and whether the groups change differently over time, mixed model approaches may be used. Comparisons between groups, time points, and the interaction of group and time will be made using linear mixed models or generalized linear mixed models, as appropriate. Mixed model analyses allow for a repeated measures approach with flexibility in variance–covariance structure while also providing estimates in the presence of potential covariates. Unadjusted and adjusted estimates will be provided by condition and time; significant group-time interactions will allow for presentation of results by condition for each time point. While the primary analyses across all aims will be the comparison of group means, the analyses for Aim 1 will additionally include the estimation of recurrent ankle sprain rates at 6, 12, and 24-months for the FIRE and SOC conditions. Moreover, time to recurrent injury will also be examined.

The primary analysis will be intent-to-treat (ITT), comparing groups as randomized. The ITT analysis will be performed using all randomized patients, where data for those terminated or lost to follow-up may be imputed using multiple imputation. If such methods are deployed, sensitivity analyses will be performed. Additional analysis will also be conducted comparing groups as they were randomized with data as observed. An attrition analysis will be conducted by comparing the demographics and outcomes measured at baseline in patients who completed follow-up to those who did not. Mechanisms for missing data will be investigated by comparing important covariates between patients with and without missing data at each time point. Furthermore, data on compliance measures will be captured within the weekly intervention logs, and a modified ITT may also be conducted but limited to those who achieved at least 75% of the intervention protocol across all six weeks. Ultimately, sensitivity analyses will be conducted comparing the results of our imputation methods to complete-case and available-data analyses. Throughout all ITT and as treated analyses, assumptions will be checked, and remedial measures will be deployed as needed.

## Discussion

CAI is a complex clinical condition associated with peripheral and central sensorimotor deficits such as cortical inhibition, peripheral deafferentation, diminished plantar cutaneous and vibration sense, and preferential shift to visual afference [7, 8, 64]. To our knowledge, this is the first clinical trial to assess the effects of IFM exercises, plantar massage, and foot stabilization exercises added to the SOC on near- and long-term functional outcomes in this clinical population. We posit that the FIRE intervention will reduce the occurrence of future ankle sprains and ankle giving way episodes and create clinically relevant improvements in sensorimotor function and self-reported disability beyond the SOC intervention alone.

Both sensory and motor mechanisms may be affected by the FIRE intervention. McKeon and Wikstrom [65] found that plantar massage improved single limb balance in individuals with CAI and postulated that the improvement was attributed to sensitization of the cutaneous plantar receptors. Similarly, the use of joint mobilization, mobilization with movement, and manipulation have been suggested to improve short-term ankle dorsiflexion motion, strength, balance, and functional test performance through both mechanical and neurophysiological mechanisms, and have been recommended for use prior to exercise in this clinical population [44]. It is highly plausible that the manual therapy interventions employed in our study will have a temporal upregulation of plantar cutaneous, muscular, and connective somatosensory receptors and central sensory modulating effect (to include motor disinhibition and mediation of nociception) that will contribute to improvements in pain, perceived stability of the ankle, balance, and patient-reported outcome measures of function.

The IFMs help to transmit or attenuate force during locomotion [66, 67]. The potential role of IFM deficits in patients with CAI has been speculated, with interventions targeting the activation, strength, and endurance of these muscles recommended as being potentially beneficial [66]. Therefore, the IFM group will be specifically targeted in our clinical trial. Two clinical trials consisting of relatively small sample sizes have examined the isolated effects of a 6-week IFM exercise program in civilian patients with CAI. Lee et al. [30] determined that the IFM exercises resulted in greater improvements in somatosensation, balance, and CAIT scores when compared to a proprioceptive exercise group. Similarly, Lee and Choi [29] identified greater increases in IFM activation and SEBT reach distances in a group of patients that completed IFM exercises compared to a control group. If these findings are generalizable to both the civilian and military populations, we anticipate that patients in the FIRE group will have significant increases in muscle activation and toe flexion strength because of the targeted interventions. We also believe that these exercises will improve somatosensation and contribute to overall improvements in balance and function.

From a methodological perspective, the decision to use an A – AB parallel design was purposeful since the experimental interventions are intended to complement the standard of care, not replace it. The comprehensive rehabilitation programs provided in both groups, which is informed by current guidelines, reflect the current standards of practice, is guided by evidence, and factors both clinician experience and patient preference [19]. This approach will ensure that the principles of equipoise, beneficence, respect for persons, justice, and the tenets of evidence-based practice are maintained for both groups. The nature of this design will also facilitate the translation of findings by providing clinicians with additional interventions to include in their current practice patterns. Knowledge products derived from the study results will include preprint archival and peer-reviewed journal submission, an evidence-based treatment protocol, and clinician training and patient education materials that will be available open access. The guidelines promulgated by the International Committee of Medical Journal Editors will be used to guide authorship decisions [68].

## Supplementary Information

**Additional file 1**. Rehabilitation guide for the supervised and home exercises utilized for the Foot Intensive Rehabilitation (FIRE) and Standard of Care (SOC) groups.

## Data Availability

Not applicable.

## Abbreviations

AP: Anterior–posterior
CA: California
CAI: Chronic ankle instability
CAIT: Cumberland ankle instability tool
CoP: Center of pressure
FABQ: Fear avoidance beliefs questionnaire
FAAM: Foot and ankle ability measure
FIRE: Foot intensive rehabilitation
ICC: Intraclass correlation coefficient
IFM: Intrinsic foot muscle
ITT: Intention to treat
LOCF: Last observation carried forward
MA: Massachusetts
MD: Maryland
mDPA: Modified disablement in the physically active scale
ML: Medial–lateral
NC: North Carolina
SEBT: Star excursion balance test
SOC: Standard of care
TTB: Time-to-boundary
VA: Virginia
