# Correction: Effects of foot intensive rehabilitation (FIRE) on clinical outcomes for patients with chronic ankle instability: a randomized controlled trial protocol

**DOI:** 10.1186/s13102-023-00679-3

**Published:** 2023-05-04

**Authors:** Matthew C. Hoch, Jay Hertel, Phillip A. Gribble, Nicholas R. Heebner, Johanna M. Hoch, Kyle B. Kosik, Doug Long, Pinata H. Sessoms, Amy Silder, Danielle M. Torp, Katherine L. Thompson, John J. Fraser

**Affiliations:** 1 Sports Medicine Research Institute, University of Kentucky, 720 Sports Center Drive, Lexington, KY 40506 USA; 2 Sports Medicine and Chair, Department of Kinesiology, University of Virginia, 550 Brandon Avenue, Charlottesville, VA 22904-4407 USA; 3 Department of Physical Therapy, College of Health Sciences, University of Kentucky, 900 South Limestone, Lexington, KY 40536-0200 USA; 4 Warfighter Performance Department, Naval Health Research Center, 140 Sylvester Road, San Diego, CA 92106-3521 USA; 5 Dr. Bing Zhang Department of Statistics, University of Kentucky, 725 Rose Street, Lexington, KY 40536 USA; 6 Naval Health Research Center, 140 Sylvester Road, San Diego, CA 92106- 3521 USA

**Correction: BMC Sports Sci Med Rehabil 15, 54 (2023)**

10.1186/s13102-023-00667-7

Following publication of the original article [1], the authors identified an omission in the current competing interests section. The competing interests should read as follows:

MCH, NRH, JMH, DMT, PHS, AS, and JJF report grants from Congressionally Directed Medical Research Programs and Office of Naval Research, outside of the submitted work. PAG, KBK, KLT report grants from Congressionally Directed Medical Research Programs, outside of the submitted work. NRH, PAG, and JMH report funding from the Federal Emergency Management Agency, outside of the submitted work. KLT reports grants from the National Institutes of Health and the National Science Foundation, outside of the submitted work. DL reports grants from the National Institutes of Health, outside of the submitted work. In addition, PHS, AS, and JJF have a patent pending for an Adaptive and Variable Stiffness Ankle Brace, U.S. Provisional Patent Application No. 63254,474.

PHS, AS, and JJF are military service members or employees of the U.S. Government and this work was prepared as part of their official duties. Title 17, U.S.C. § 105 provides that copyright protection under this title is not available for any work of the U.S. Government. Title 17, U.S.C. § 101 defines a U.S. Government work as work prepared by a military service member or employee of the U.S. Government as part of that person’s official duties. The views expressed in this article are those of the authors and do not necessarily reflect the official policy or position of the Department of the Navy, Department of Defense, nor the U.S. Government. The study protocol was approved by the University of Kentucky Institutional Review Board in compliance with all applicable Federal regulations governing the protection of human subjects, number 58,500.
