# Supplementary material for: Effects of foot intensive rehabilitation (FIRE) on clinical outcomes for patients with chronic ankle instability: a randomized controlled trial protocol
Source: BMC Sports Sci Med Rehabil. 2023 Apr 9;15:54. doi: 10.1186/s13102-023-00667-7 (PMC10084629; doi:10.1186/s13102-023-00667-7)

FIRE SUPERVISED INTERVENTION PROTOCOL

The exercises outlined below are associated with the FIRE intervention arm of this RCT. Subjects allocated to the SOC group should follow the *SOC Supervised Intervention Protocol*. This protocol is broken down into 5 target areas (Ankle ROM, Ankle/Hip Strength, Static Balance, Dynamic Balance, and Sensorimotor Foot). Exercises for each target area will be performed during each supervised rehabilitation session and documented in the log. **Exercises should be performed in the order as presented in the FIRE Supervised Session Log.**

- The log should document the progression and volume of the exercise completed following each session.
- All subjects are asked to complete two supervised rehabilitation sessions per week for all six weeks.
- Care should be taken to observe exercises included in the home exercise program to ensure they are performed correctly and completely to facilitation the unsupervised home sessions.
- Day 1 Intervention: establish starting levels for each exercise.

I. Foot Intensive Rehabilitation (FIRE) Protocol

Goal #1: Improve Ankle Range of Motion

a. Talocrural Joint Mobilization

2 x 2-minute sets of Maitland Grade III anterior-to-posterior talocrural joint mobilizations with 1 minute rest between sets throughout the sessions. Begin with a distraction of the foot and apply oscillations from mid-range to end-range of accessory motion over the course of one second.

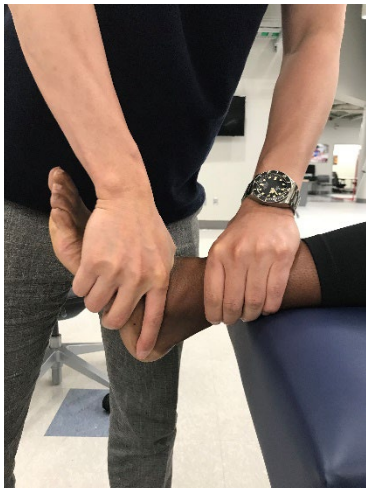

b. **Heel cord stretching**

Patients will be instructed on two variations of heel cord stretches to target the gastrocnemius and the soleus. Progressions will be created through tandem stance, foot on wall, and slant board variations. Dosage is 3 sets of 30 seconds for each condition (knee straight, knee bent) and alternating between front and back leg stretching.

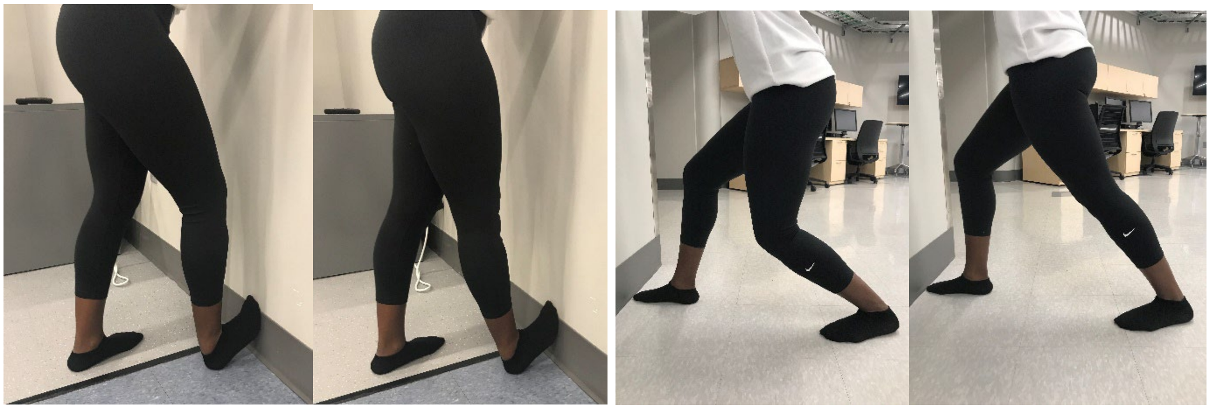

c. **Wobble Board**

While seated with a wobble board placed in front of the patient so their involved foot is placed comfortable in the middle of the board and tibia perpendicular to the floor. The patient is instructed to rotate the ankle with the goal of touching the edges of the wobble board to the ground. Cues are controlled motion, quiet, and smooth rotations.

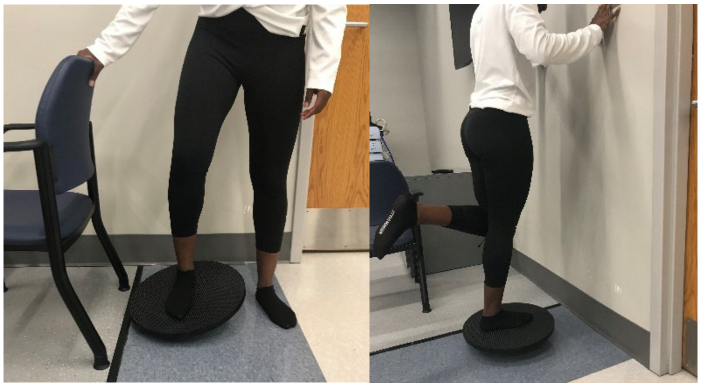

<table>
<tr><th>Exercise</th><th>Progression</th><th>Position</th><th>Volume</th><th>Progress</th></tr>
<tr><td rowspan="6">Only do one direction each session (e.g, CW day 1/ CCW day 2)</td><td>Level 1</td><td>Seated</td><td>3 x 5</td><td rowspan="6">when patient performance is controlled and does not feel challenged.</td></tr>
<tr><td>Level 2a</td><td>DBL stance</td><td>2x5</td></tr>
<tr><td>Level 2b</td><td>DBL Stance</td><td>2x10</td></tr>
<tr><td>Level 2c</td><td>DBL stance</td><td>2x15</td></tr>
<tr><td>Level 3a</td><td>SL Stance (wall support)</td><td>2x5</td></tr>
<tr><td>Level 3b</td><td>SL Stance (wall support)</td><td>2x10</td></tr>
</table>

Goal #2: Improve Ankle/Hip Strength

a. **Ankle Isotonics with Resistance Band**. Resistive band supination or pronation in long sitting position. Patient is instructed to avoid allowing the band to pull them back to the starting the position ie “you control the motion. Don’t allow the band to control it.” The band should be stretched to 70% of its resting length and movement through the range of motion should occur at a consistent pace of approximately 3 to 5 seconds per repetition throughout the full range of motion.

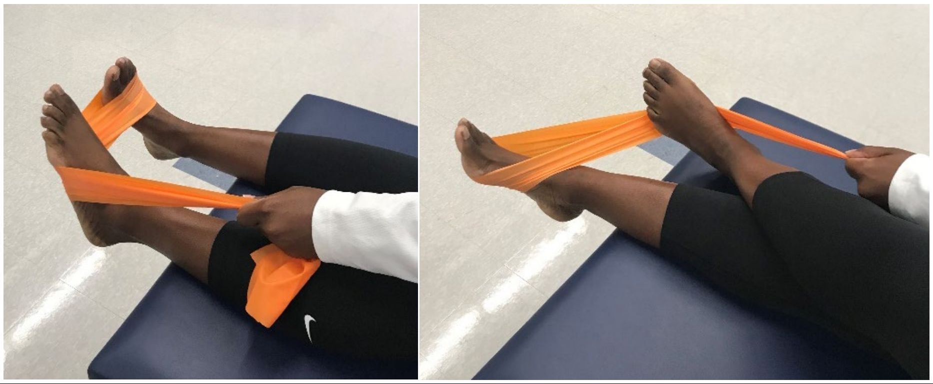

Level	Color	Volume	Progress to next level every two weeks
Level 1	Green	2 x 10	
Level 2	Blue	2 x 10	
Level 3	Black	2 x 10	

b. **Double-Leg heel raises with ball squeeze**

While standing near a chair or wall for assistance with balance as necessary, the patient stands on both limbs and lifts the heels up to maximum plantarflexion while squeezing a ball placed between the heels (targeting posterior tibialis), and then lowers in the same slow and controlled manner. This task will be progressed to double-leg heel raises off a step with patient’s forefoot on an elevated platform or stair to allow for increased range of motion. Patient is instructed to emphasize a slow and controlled lowering/eccentric phase.

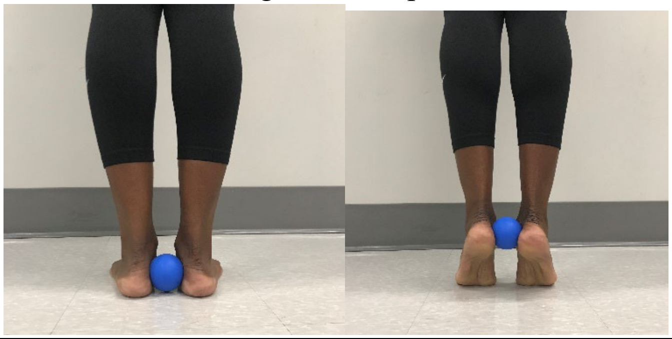

Level	Position	Volume	Progress to next level when movement is controlled, and pt is not challenged.
Level 1a	Floor	3 x 10	
Level 1b	Floor	3 x 15	
Level 2a	Box	2 x 10	
Level 2b	Box	2 x 15	
Level 2c	Box	2 x 20	

c. Closed Chain Resisted Foot Adduction

Patients are seated with their knees maintained at a forearm's length apart and flexed approximately 80°, with feet on the ground. The patient is asked to stabilize their leg by placing the contralateral forearm between the knees and reinforcing it with the ipsilateral hand. An elastic band is looped around the distal and medial foot being exercised. The elastic band is stretched laterally to full tension, while maintaining a 45° angle of inclination with the floor. The investigator holds the band with the goal of maintaining constant tension throughout completion of this exercise. From an abducted position, the patient will slide their forefoot into adduction and then slowly returned to the starting position. Each subject's individual foot range of motion in the transverse plane was marked on the floor and that range was achieved with each repetition. The foot remained flat on the floor during the entire exercise.

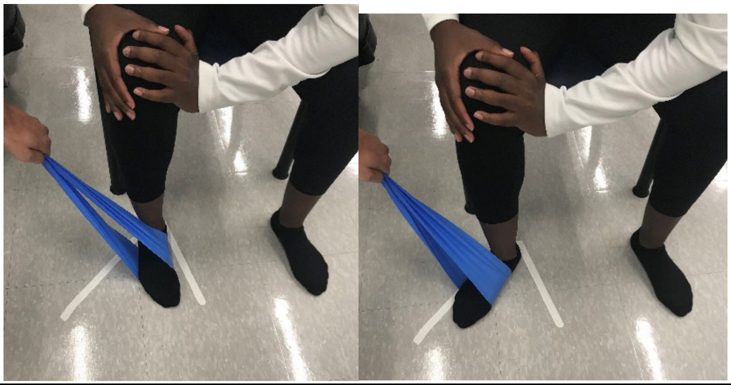

Level	Color	Volume	Progress to next level when movement is controlled, and pt is not challenged.
Level 1	Green	2 x 10	
Level 2	Blue	2 x 10	
Level 3	Black	2 x 10	

d. Single-leg Kicks (Steamboats)

While standing on the involved limb and a light resistance band anchored low and placed around the distal leg, the patient moves against the band and returns to start. The motion is 1-second count, controlled return (eccentric) and the patient avoids holding onto anything for balance.

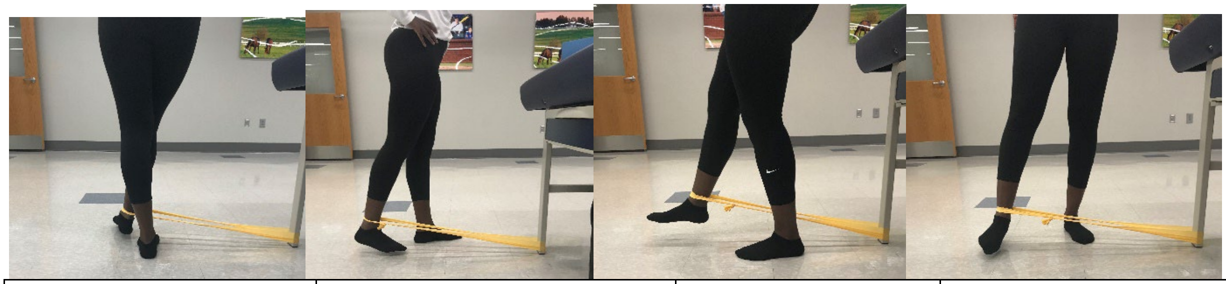

Level	Color	Volume	Progress to next level when movement is controlled, and patient is not challenged.
Level 1a	Green	2 x 10	
Level 1b	Green + Foam Pad	2 x 10	
Level 2a	Blue	2 x 10	
Level 2b	Blue + Foam Pad	2 x 10	
Level 3a	Black	2 x 10	
Level 3b	Black + Foam Pad	2 x 10	

e. **Rotational Lunge**

Set Up: Patient sits on floor with legs extended heels touching a wall. A mark is place on floor to show the leg-length, which will be the near-foot starting point for the exercise.

Task: Patient will stand with both feet parallel, with the lateral border of the near-foot at the previously placed mark. The patient will be instructed to pick up that foot, externally rotate 90 degrees and lunge with the toes and knee pointing towards the wall (perpendicular to starting position). Where the patient is able to touch the wall with the knee (60-90 degrees of knee flexion), an additional mark is placed on the wall to give the patient a point of reference for consistency. The end of the repetition is the return to the starting position. The patient is instructed to consider a 4-count execution to aid in consistent and controlled movement:

1. Move from starting position to foot placement
2. Lunge forward (knee flexes and touches wall)
3. Knee extends and begin return to start
4. Return to starting position

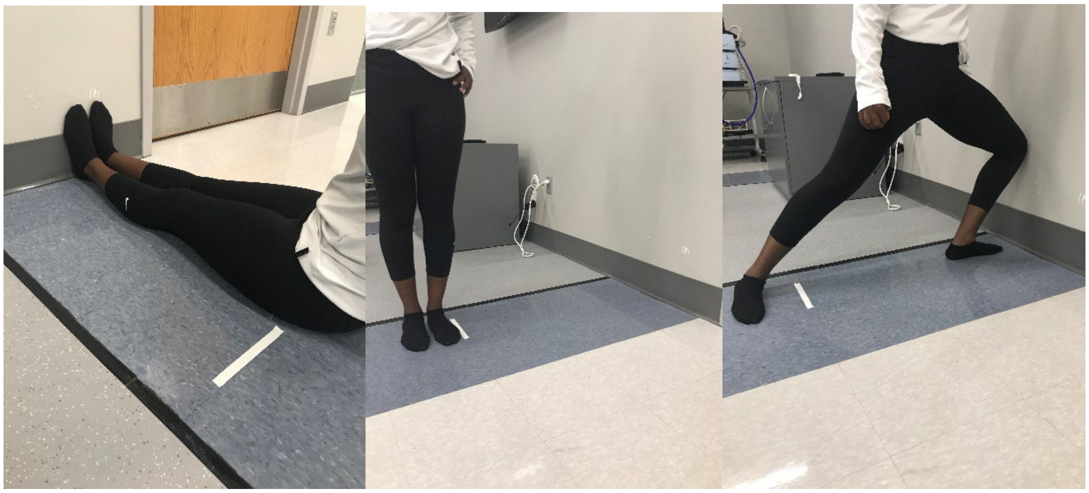

Level	Position	Volume	Progress to next level when movement is controlled, and pt is not challenged.
Level 1a	Floor	2 x 10	
Level 1b	Floor	2 x 15	
Level 1c	Floor	2 x 20	
Level 2a	Foam Pad (at wall)	2 x 10	
Level 2b	Foam Pad (at wall)	2 x 15	
Level 2c	Foam Pad (at wall)	2 x 20	

f. **Rotational Squat**

Set Up: The patient stands arms distance (tip of 3rd digit in contact with the wall) and a mark is place on the wall at the height of the patient's lateral femoral condyle.

Task: Patient will stand on the near-limb, squat down and rotate laterally, placing stance hip in internal rotation, and reach towards the target with both hands (but is permitted to concentrate on the near hand). The end of the repetition is the return to the starting position, remaining on one limb for the entire exercise. They may place to other foot down in between repetitions. The patient is instructed to consider a 4-count execution to aid in consistent and controlled movement:

1. Move from starting position to single leg squat
2. Rotate to wall and tap target
3. Rotate back to neutral hips, single leg-squat
4. Return to starting position

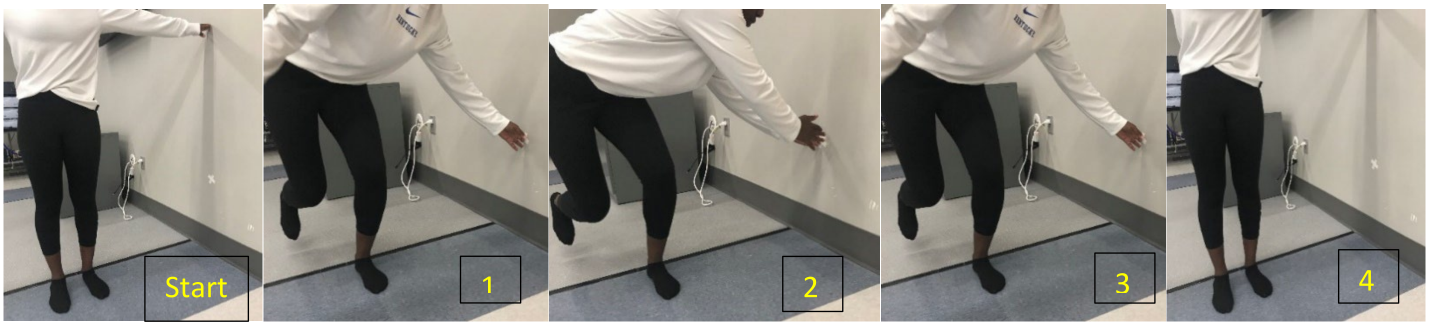

Level	Position	Volume	Progress to next level when movement is controlled, and pt is not challenged.
Level 1a	Floor	2 x 10	
Level 1b	Floor	2 x 15	
Level 1c	Floor	2 x 20	
Level 2a	Foam Pad (at wall)	2 x 10	
Level 2b	Foam Pad (at wall)	2 x 15	
Level 2c	Foam Pad (at wall)	2 x 20	

Goal #3: Improve Static Balance

a. **Single Limb Balance Progression - Eyes Open**

Patients will complete a single limb balance progression which incorporates changes in visual status, stance surface, and stance duration. However, to be considered for progression, the patient must not exhibit any errors (rather than progressing off of time alone.)

Errors are the same as for the Balance Error Scoring System: taking one or both hands off the hips, touching down with the opposite limb, trunk flexion or lean greater than 30 degrees.

For progression on stable surfaces, the patient must not exhibit any errors and maintain intrinsic foot muscle activation for at least half the trial. This will be observed by examining motion of the navicular tuberosity and medial longitudinal arch contact with the ground.

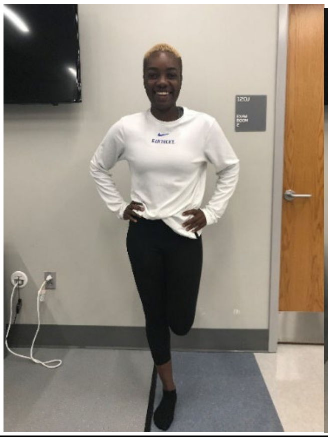
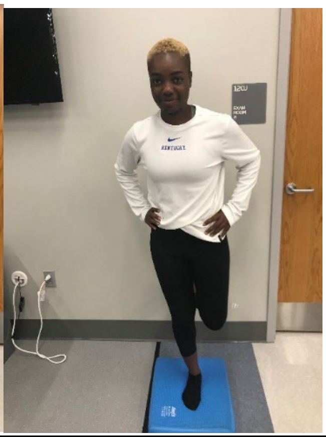

Eyes Open			
Level	Position	Volume	Progression when no errors (see above)
Level 1	Floor	3 x 60s	
Level 2a	Foam Pad	3 x 30s	
Level 2b	Foam Pad	3 x 60s	
Level 2c	Foam Pad	3 x 90s	

b. **Single Limb Balance Progression - Eyes Closed**
Patients will complete a single limb balance progression which incorporates changes in stance surface, and stance duration. However, to be considered for progression, the patient must not exhibit any errors (rather than progressing off of time alone.)

Eyes Closed			
Level	Position	Volume	Progression when no errors (see description above)
Level 1a	Floor - arms out	3 x 30s	
Level 1b	Floor – arms across chest	3 x 30s	
Level 1c	Floor – arms across chest	3 x 60s	
Level 2a	Foam pad – arms out	3 x 30s	
Level 2b	Foam pad – arms across chest	3 x 30s	
Level 2c	Foam pad – arms across chest	3 x 60s	
Level 2d	Foam pad – arms across chest	3 x 90s	

Goal #4: Improve Dynamic Balance

a) Hop to Stabilization

The patient hops 18 inches anteromedial, stabilizes briefly, and then hops back to starting position. The movement is repeated to the anterolateral direction and back. The patient should be positioned forward through each trial. Progressions will be made to the next hop distance when the participant can complete the hop series error-free.

Anterolateral:

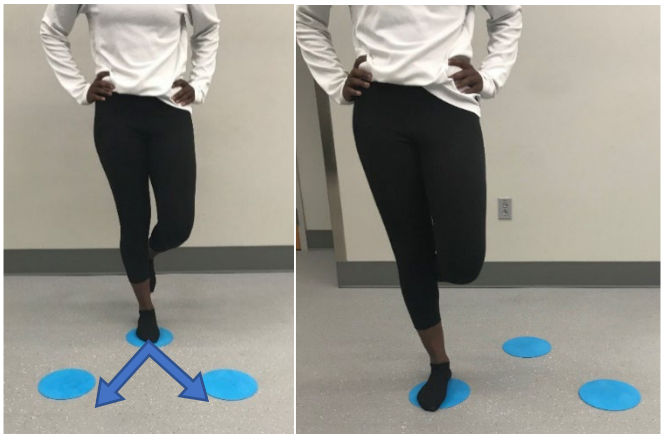

Anteromedial:

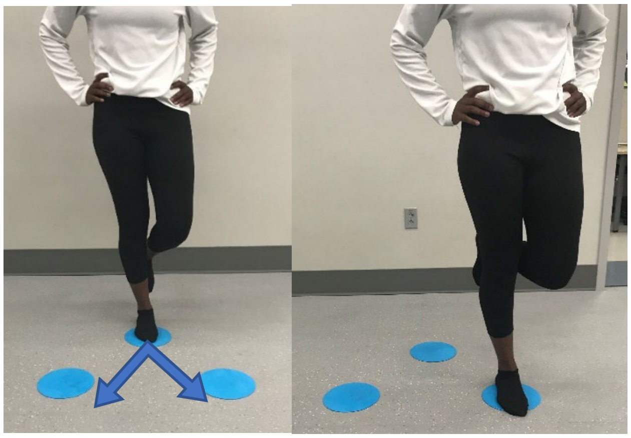

Anteroposterior:

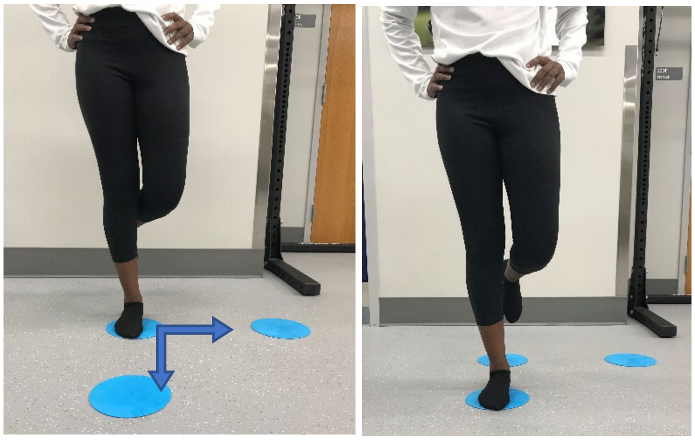

Mediolateral:

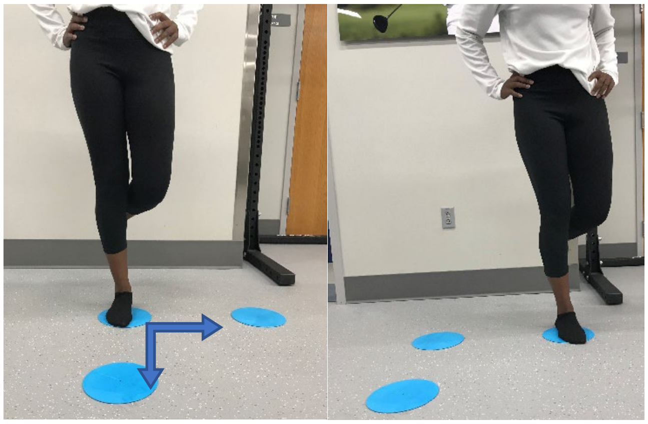

Level	Position	Volume (per direction)	Progress to next level when error-free in each direction
Level 1	18 in – Arms out	1 x 10	
Level 2	18 in – Hands on hips	1 x 10	
Level 3	27 in – Arms out	1 x10	
Level 4	27 in – Hands on hips	1 x 10	
Level 5	36 in – Arms out	1 x 10	
Level 6	36 in – Hands on hip	1 x 10	
Level 7	36 in – from 6in platform	1 x 10	

b) Hop to Stabilization and Reach

This task is similar to the Hop to Stabilization task but after stabilization in the single-limb stance, participants had to reach back to the starting position, hop back to the starting position, and then reach to the target position. Participants were not able to advance to the next level in each direction until they demonstrated five repetitions error-free while maintaining hands on hips.

Anterolateral:

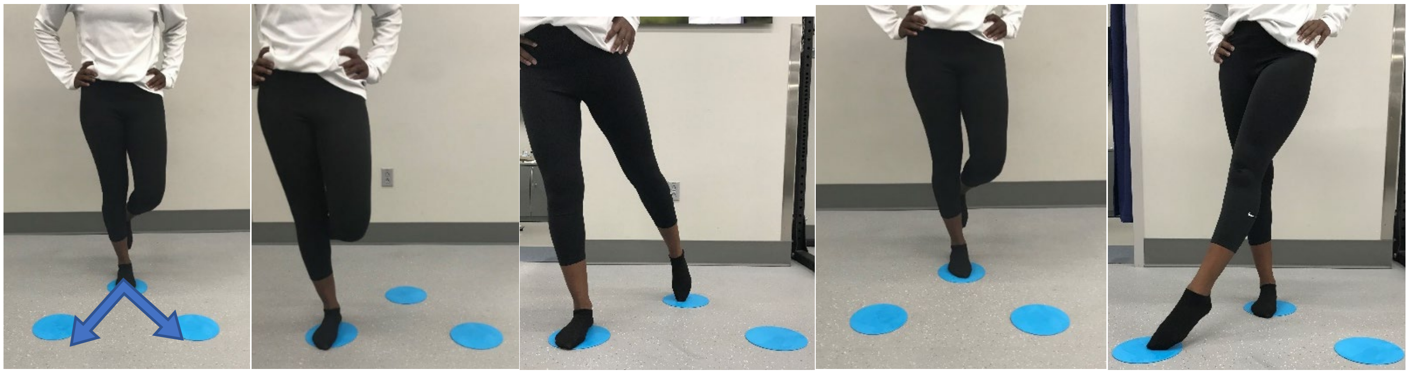

Anteromedial:

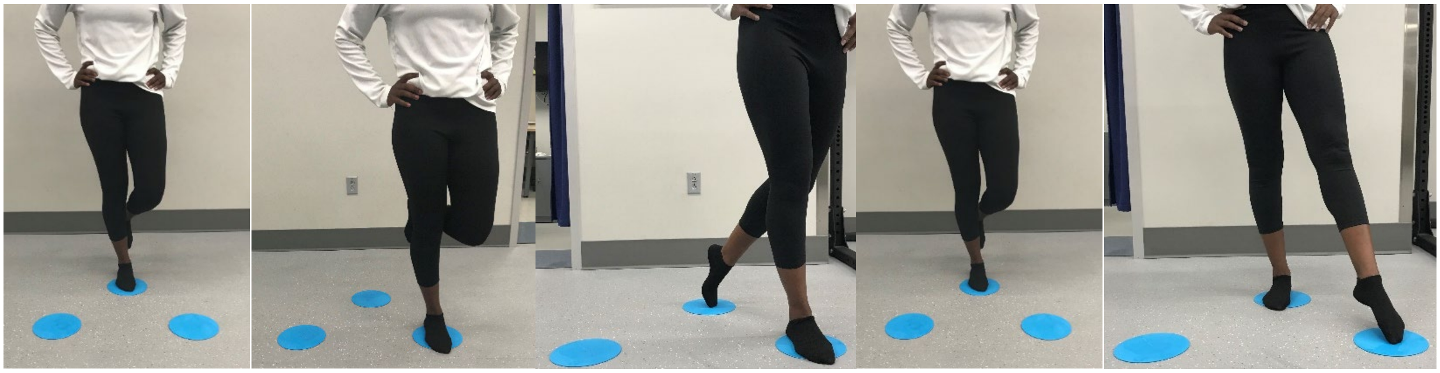

Anterior-Posterior:

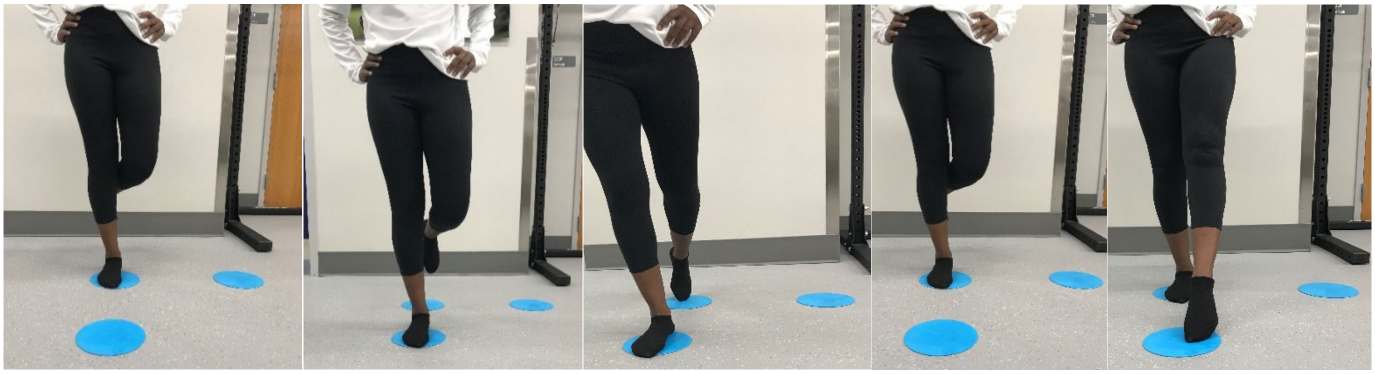

Medial - Lateral

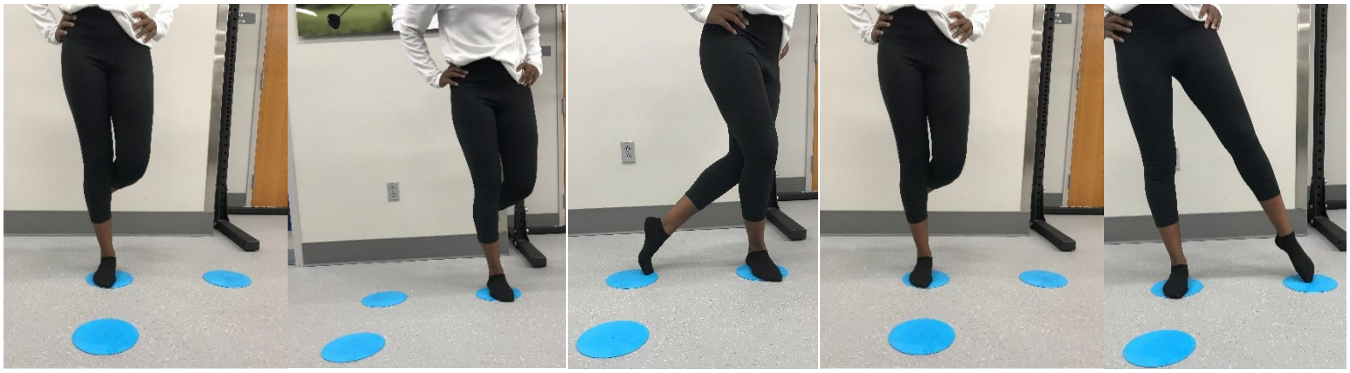

Level	Position	Volume (per direction)	Progress to next level when error-free in each direction
Level 1	18 in – Arms out	1 x 10	
Level 2	18 in – Hands on hips	1 x 10	
Level 3	27 in – Arms out	1 x 10	
Level 4	27 in – Hands on hips	1 x 10	
Level 5	36 in – Arms out	1 x 10	
Level 6	36 in – Hands on hip	1 x 10	
Level 7	36 in – from 6in platform	1 x 10	

c) Unanticipated Hop to Stabilization

With a grid on the floor (numbered rubber discs are ideal for this) of 9 markers placed 18 inches apart, the clinician will call out numbers in a random order, but adjacent to the number the patient is standing on. The goal is 3 sets of 10 hops allowing 5 seconds to complete each hop, progressing to a 3 second time limit, and eventually a 1 second time limit. When progression indicates addition of a foam pad(s), place numerical dot on top of pad (sandwich) to maintain position and give patient a target.

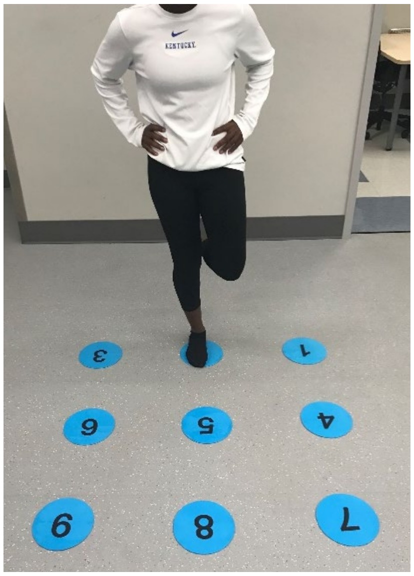

The errors for all hop tasks included:

- Touching down with opposite limb
- Excessive trunk motion (30°- lateral flexion)
- Removal of hands from hips during hands on hips activities
- Bracing the nonstance limb against the stance limb
- Missing the target

The premise for these tasks is the same as described in SOC protocol. However, for progression, the patient must not exhibit any errors and maintain intrinsic foot muscle activation following stabilization. This will be observed by examining motion of the navicular tuberosity and medial longitudinal arch contact with the ground.

Errors and progression criteria should be followed based on the appendix material in McKeon 2008 Med Sci Sport Exer

<table>
<tr><th>Level</th><th>Position</th><th>Volume</th><td rowspan="5">Progress to next level when error-free; progress each direction individually</td></tr>
<tr><td>Level 1</td><td>5 seconds (a)</td><td>3 x 10</td></tr>
<tr><td>Level 2</td><td>3 seconds (b)</td><td>3 x 10</td></tr>
<tr><td>Level 3</td><td>1 second (c)</td><td>3 x 10</td></tr>
<tr><td>Level 4a-c</td><td>Add foam pad to one number</td><td>3 x 10</td></tr>
</table>

Goal #5: Improve Sensorimotor Function of the Foot

a. **Plantar cutaneous massage***
Manual massage (combination of effleurage and petrissage) at dosage of 2 sets of 1 min, with 1 minute of rest between sets. The goal should be to provide massage to all plantar foot surfaces evenly during each set.

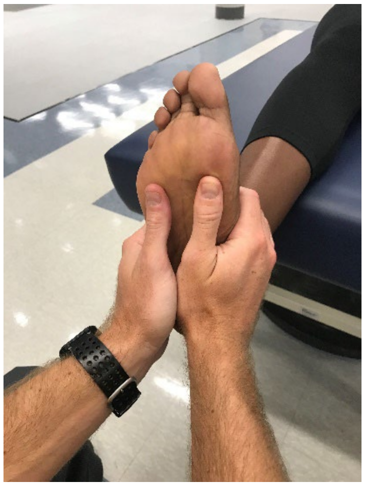

b. **Intrinsic Foot Muscle Activation**

Four exercises will target the IFMs: A) short-foot, B) toe-spread-out, C) hallux extension, and D) lesser-toe extension. These exercises have demonstrated ability to activate the IFMs. In the first treatment session, subjects will start each exercise in a seated position. Progression to double-limb stance and single-limb stance will occur when an exercise is done correctly for an entire session without compensation. The number of repetitions, contraction durations, and progressions were adapted from Fraser and Hertel[10] and are further outlined in the Intervention attachment.
Intrinsic Foot Muscle Exercises

A. The short foot exercise will involve drawing the metatarsal heads towards the calcaneus while doming the medial longitudinal arch without extrinsic muscle compensation.
B. Hallux extension exercise will be completed by extending the great toe while toes 2-5 remained on the floor in a neutral position.
C. The toe-spread-out exercise will be performed by extending the toes and then simultaneously abducting the toes and flexing the first and fifth toe to the ground while toes 2-4 remain extended. The middle toes are then relaxed.
D. Lesser-toe extension will be completed by extending toes 2-5 while the great toe remains flat.

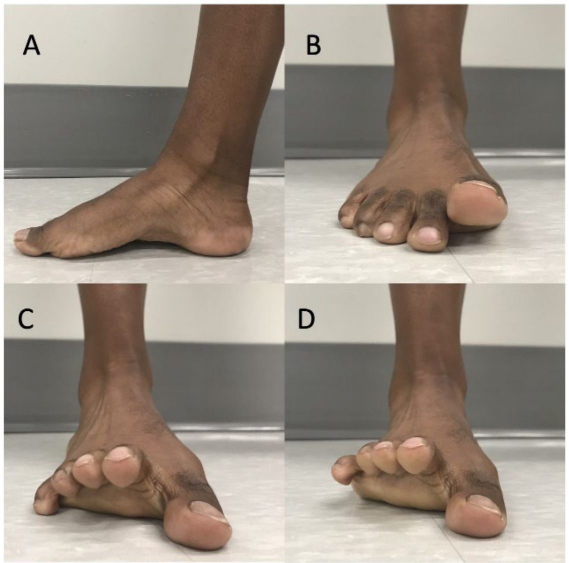

Exercise	Progression	Position	Day 1	Day 2
Short Foot<br>Hallux Ext<br>Lesser Ext<br>Toe-Spread	Level 1	Seated	2 x 15 @ 3s<br>2 x 8 @ 8s	2 x 8 @ 8s<br>2 x 3 @ 20s
	Level 2	DBL stance	2 x 15 @ 3s<br>2 x 8 @ 8s	2 x 8 @ 8s<br>2 x 3 @ 20s
	Level 3	SL Stance	2 x 15 @ 3s<br>2 x 8 @ 8s	2 x 8 @ 8s<br>2 x 3 @ 20s

c. **Step-Up with Active Supination or Pronation**
The patient will be instructed to step up onto a platform with either the lateral or medial border of the foot suspended off the edge of the step. While performing the step up maneuver the patient will actively elevate the suspended portion of the foot. The patient will be cued to make the suspended part of the foot "level" with remaining aspects of the foot on the step during the task.

Supination- Standing with support

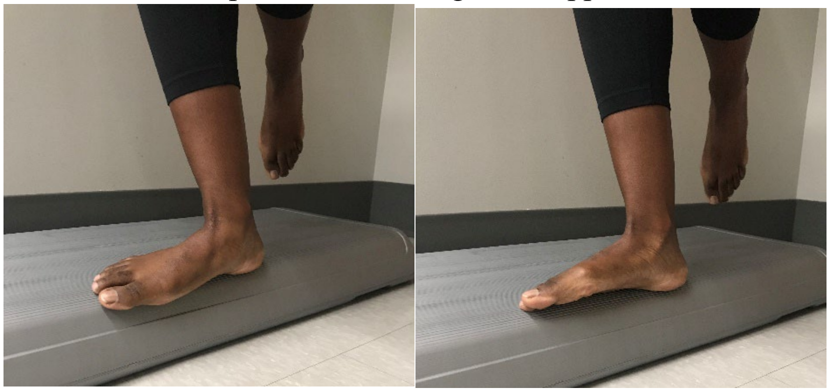

Supination with step-up

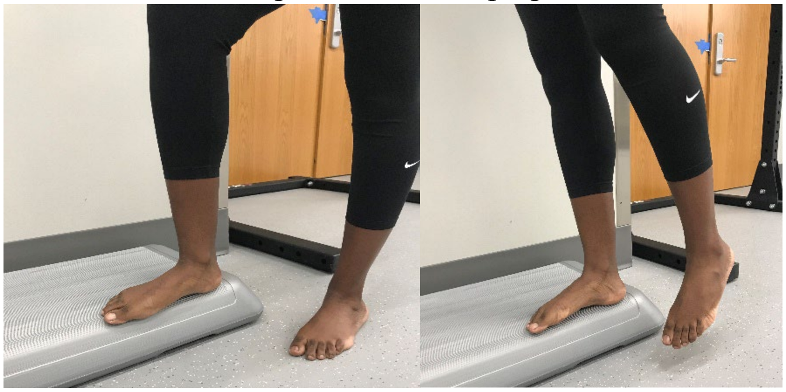

Pronation – Standing with Support

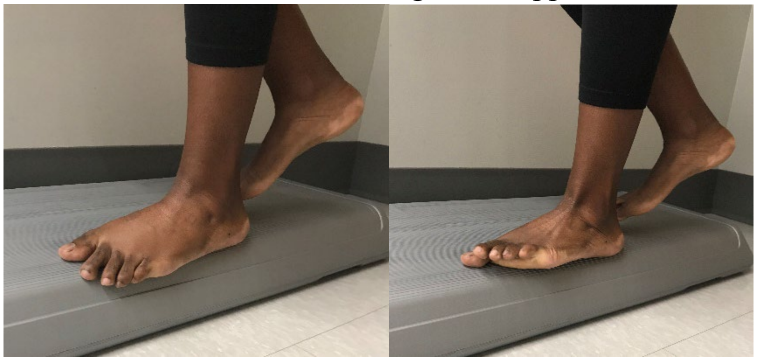

Pronation with step-up

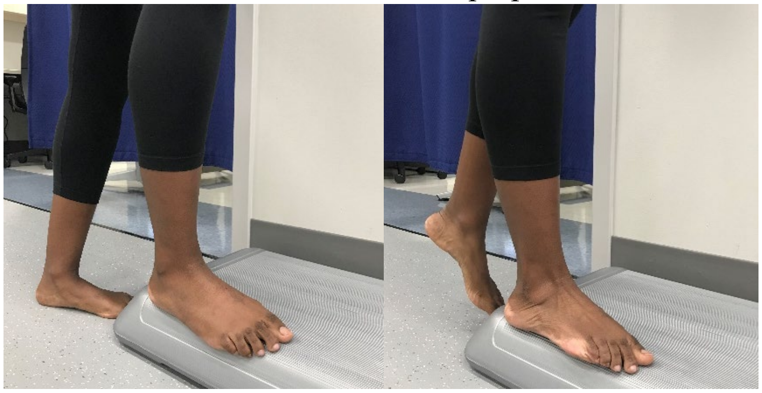

Level	Position	Step Height	Volume	Progression when patient is stable when performing task and maintaining foot level
Level 1a	Standing with support	10 cm	2 x 10	
Level 1b	Standing with support	10 cm	2 x 15	
Level 2a	Step Up	20 cm	2 x 10	
Level 2b	Step Up	20 cm	2 x 15	
Level 3a	Step Up	30 cm	2 x 10	
Level 3b	Step Up	30 cm	2 x 15	

Foot Intensive Rehabilitation Home Exercise Library

This library is your reference for the exercises that may be included in your home exercise plan. You may or may not be prescribed all of these exercises for each session. Please refer to your Home Exercise Prescription or contact your provider with questions.

Calf Stretching Option 1 (front leg is stretching)

Stand near a wall with the forefoot of you injured foot against the wall. Lean forward with your knee straight until you feel a gentle stretch in our calf muscle and calf. Return to the starting position and repeat with the front knee bent.

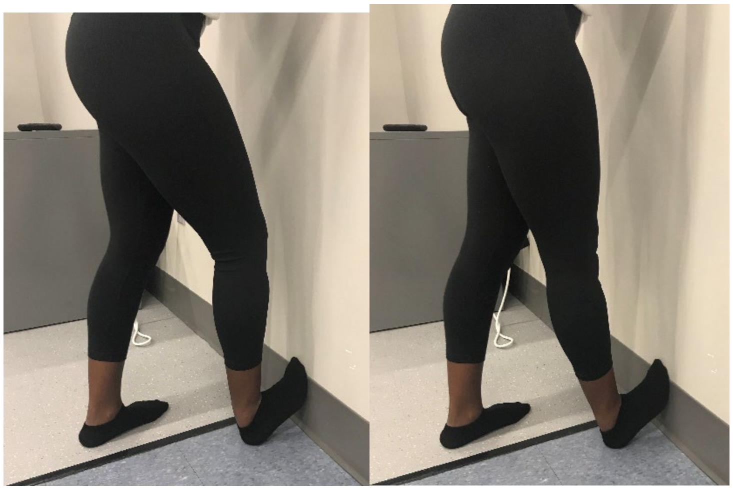

Calf Stretching Option 2 (Back leg is stretching)

Stand with feet shoulder width apart and your non-injured foot forward, with both feet pointing straight ahead. Keep back leg straight and lean forward, bending the knee of your front leg. Make sure you keep your heels flat on the floor. Drift forward until you feel a pull behind your knee or calf and hold.   Return to starting position, and repeat with the back leg bent.

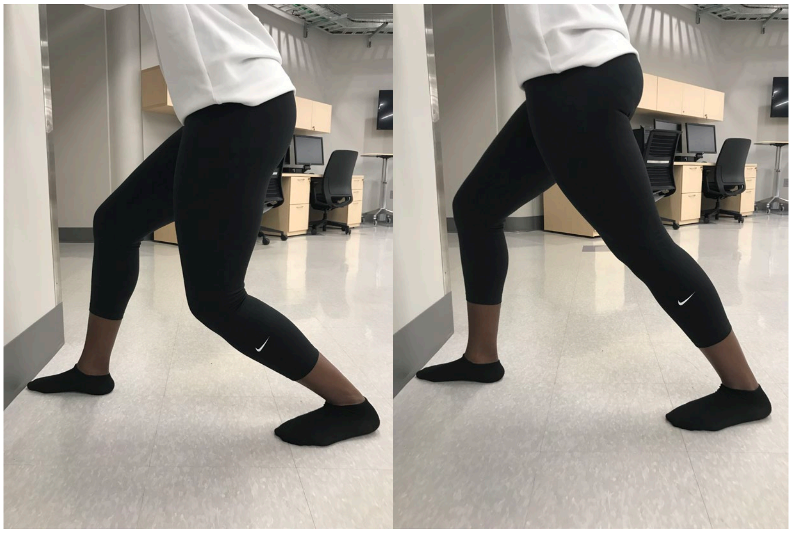

Resistive Band Exercises

All resistance band exercises are slow and controlled. Do not let the band "bounce" back. When you move to the end of the motion, hold the contraction for 3 seconds. Additionally, try to replicate the resistance of the band that you are experiencing during supervised rehabilitation sessions.

A. Pronation: Keep both knees straight. Place the loop of the band around "outside border of foot" and press it away as far as possible, hold for 3 sec, and slowly return to neutral. Repeat.

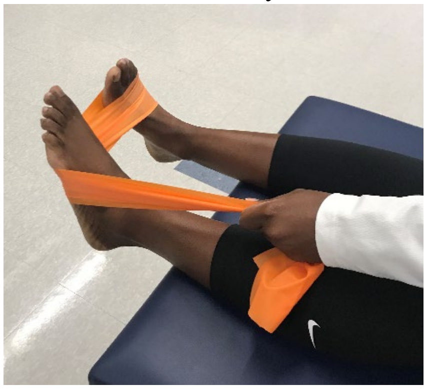

B. Supination: Keep the knee of the ankle you are exercising straight. Cross the opposite leg over top and use the foot as a pivot point for the resistance band. Place the loop of the band around "ball of foot and big toe" and press it away as far as possible, hold for 3 sec, and slowly return to neutral. Repeat.

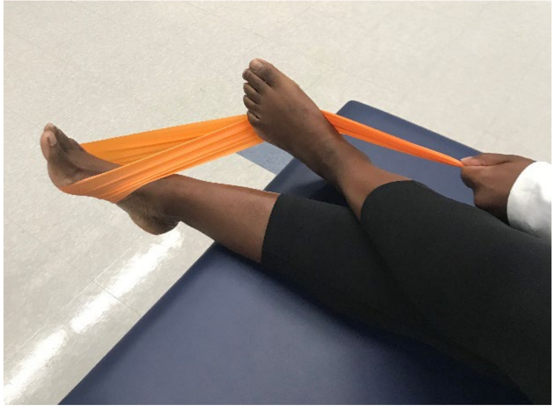

Heel Raise with Ball Squeeze

While standing on both legs, raise up on your toes as you lift your heel off the ground. Squeeze a ball between your heels while raising and lowering to the ground. Do this next facing a wall in case you lose your balance, but try not to use the wall. You should raise and lower your heels in a slow and controlled movement. Repeat.

This exercise will be progressed by placing your toes and the ball of your foot on the edge of the foam pad used for the unstable balance exercises and performing the same motion.

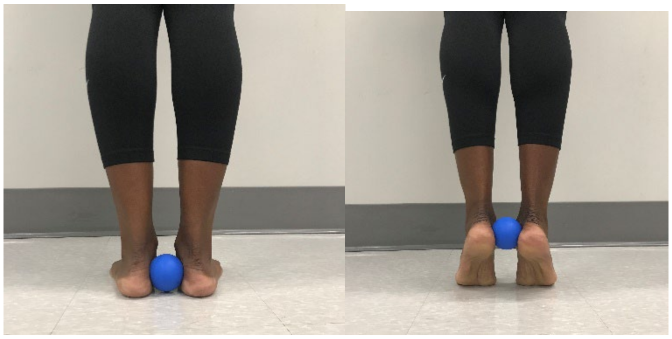

Single Leg Balance

Practice balancing on your non-injured leg (barefoot), keeping your hands on your hips. Once you can balance for 20 seconds with your eyes open, practice with eyes closed. Tip: find your balance first with your eyes open. Then practice on a non-stable surface using the balance pad provided.

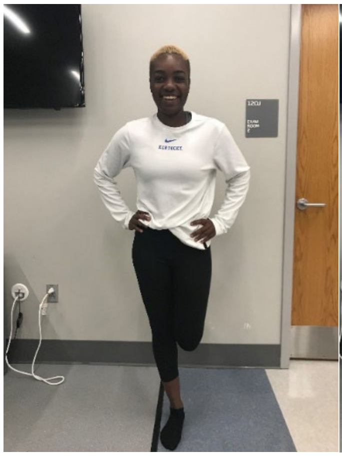
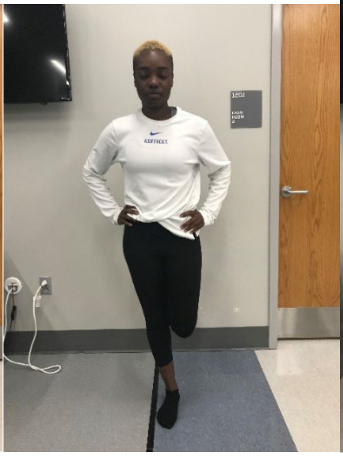
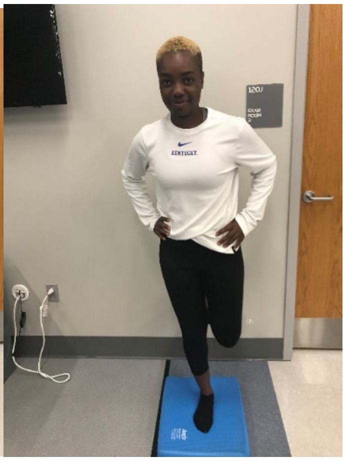

Side-Lying Glute Strengthening
While lying on your side, slowly raise up your top leg to the side. Keep your knee straight and your toes pulled up towards your shin the entre time. Keep your leg in line with your body and your hips stacked; do not let your torso roll forward or back. The bottom leg should be bent to stabilize your body. Hold this position for 3 sec and then slowly lower your top leg. This exercise will be progressed by adding a resistance band around both thighs.

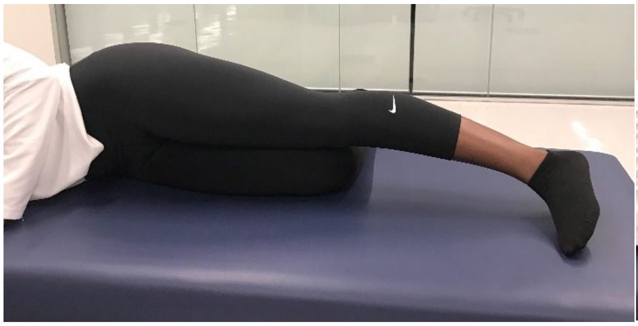
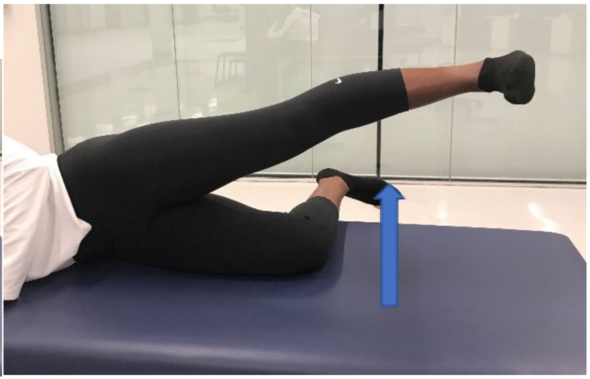

Prone Leg Extension While lying face down with your knee bent, slowly raise your knee off the ground. Try to keep both front hip bones in contact with the ground and avoid twisting at your waist.

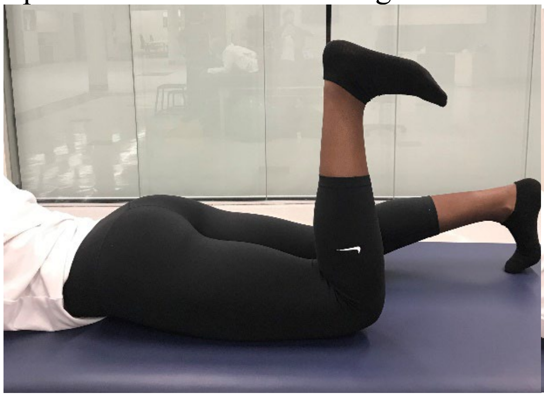
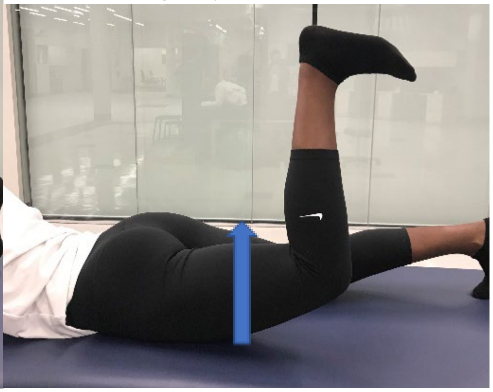

Rotational Squat

Place a post-it note or piece of tape on the wall at the level of the bony prominence below your knee cap (tibial tuberosity). Stand one arm's length away from the wall with the injured leg side closest to the wall. This move is four-count. 1) On the leg closest to the wall, squat down while keeping your hips facing forward. 2) Rotate towards the wall and tap your mark with the closest hand. If you cannot reach it, you most likely need to squat lower next time. 3) Return to facing forward. 4) Straighten your knee to return to standing.

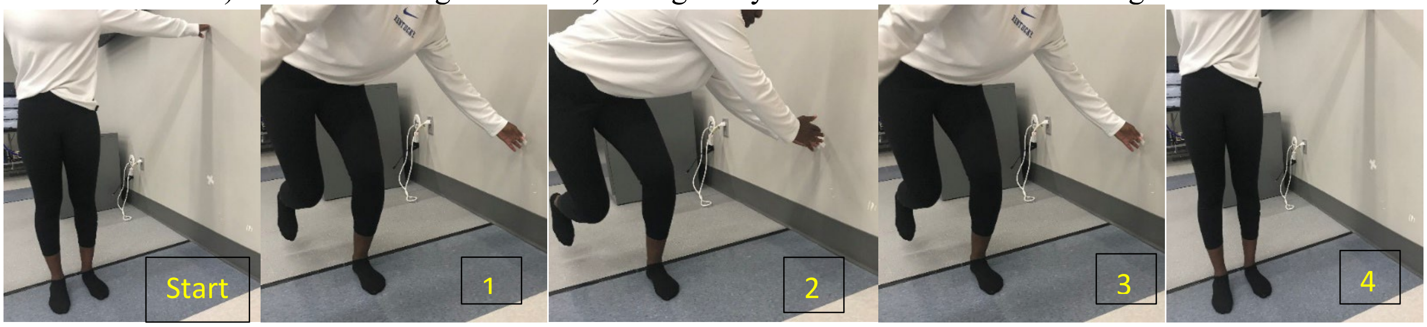

Rotational Lunge

Using the same mark on the wall as the rotational squat, start by standing parallel to the wall one leg's distance from the wall. Stand so that your injured side is closest to the wall. Step towards the wall, and bend that leg to tap your mark on the wall with the front of your knee. Return to staring position.

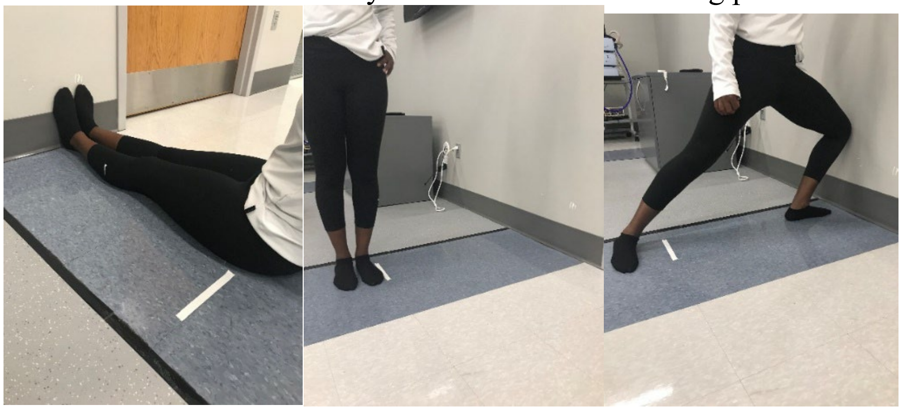

Massage

While seated, use your massage ball to roll the bottom of your feet. Make an imaginary grid (up and down, side to side) to cover the entire surface. It doesn't have to hurt to be effective: use a comfortable amount of pressure.

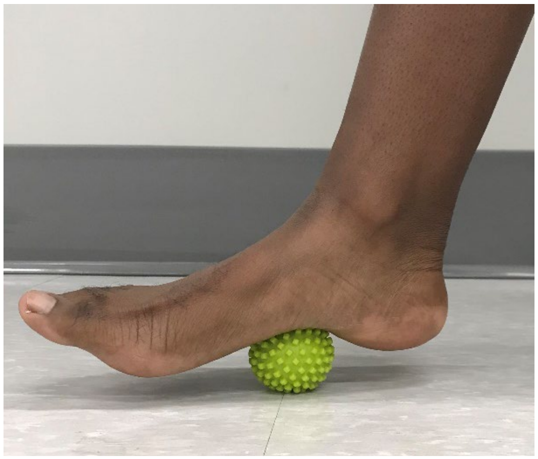

Foot Muscle Exercises

Four exercises will target the smaller muscles that are on the bottom of your feet. The goal of these exercises is to focus on controlling specific muscles in your foot that result in movement of the foot arch or toes. It may take a few sessions of working with your supervising clinician to gain control over these movements. Over the course of rehabilitation you may complete these while sitting, standing on two legs, or standing on a single leg. In each position, the goal is to slowly create the contraction, hold it for the specified amount of time, and slowly return to the starting position. Contraction times for these exercises are noted as either 3, 8, or 20 seconds.

A. The short foot exercise will involve drawing the ball of your foot towards your heel while doming the arch.
B. Hallux extension exercise will be completed by raising the great toe from the floor while toes 2-5 remain on the floor in a neutral position.
C. The toe-spread-out exercise will be performed by raising the toes and then simultaneously spreading the toes and lowering the first and fifth toe to the ground while toes 2-4 remain raised. The middle toes are then relaxed.
D. Lesser-toe extension will be completed by raising toes 2-5 from the floor while the great toe remains flat.

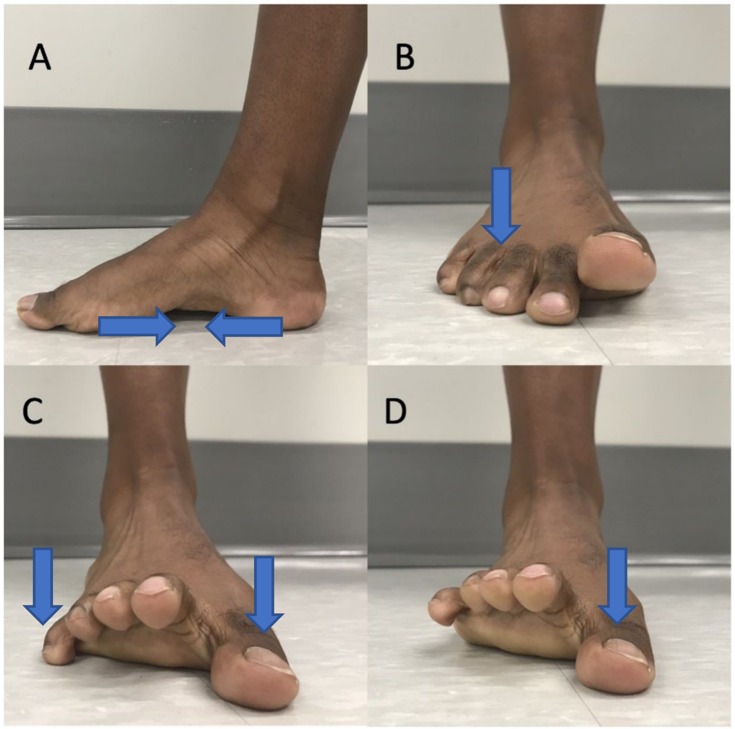

SOC SUPERVISED INTERVENTION PROTOCOL

The exercises outlined below are associated with the SOC intervention arm of this RCT. Subjects allocated to the FIRE group should follow the *FIRE Supervised Intervention Protocol*. This protocol is broken down into 4 target areas (Ankle ROM, Ankle/Hip Strength, Static Balance, and Dynamic Balance). Exercises for each target area will be performed during each supervised rehabilitation session and documented in the log.

- The log should document the progression and volume of the exercise completed following each session.
- All subjects are asked to complete two supervised rehabilitations sessions per week for all six weeks.
- Care should be taken to observe exercises included in the home exercise program to ensure they are performed correctly and completely to facilitation the unsupervised home sessions.
- Day 1 Intervention: establish starting levels for each exercise.

I. Standard of Care (SOC) Protocol

Goal #1: Improve Ankle Range of Motion

a. Talocrural Joint Mobilization
2 x 2-minute sets of Maitland Grade III anterior-to-posterior talocrural joint mobilizations with 1 minute rest between sets throughout the sessions. Begin with a distraction of the foot and apply oscillations from mid-range to end-range of accessory motion over the course of one second.

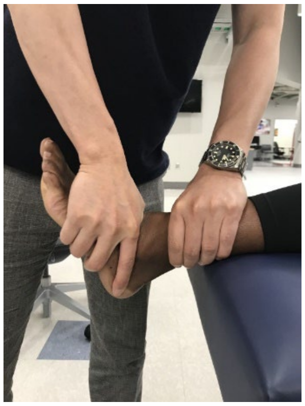

b. Heel cord stretching
Patients will be instructed on two variations of heel cord stretches to target the gastrocnemius and the soleus. Progressions will be created through tandem stance, foot on wall, and slant board variations. Dosage is 3 sets of 30 seconds for each condition (knee straight, knee bent.)

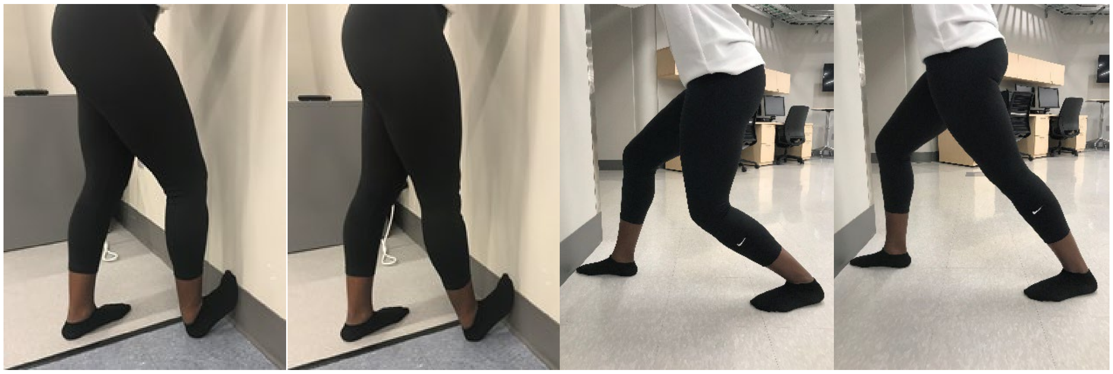

c. **Wobble Board**

While seated with a wobble board placed in front of the patient so their involved foot is placed comfortable in the middle of the board and tibia perpendicular to the floor. The patient is instructed to rotate the ankle with the goal of touching the edges of the wobble board to the ground. Cues are controlled motion, quiet, and smooth rotations.

Exercise	Progression	Position	Volume	Progress
Only do one direction each session (e.g, CW day 1/ CCW day 2)	Level 1	Seated	3 x 5	when patient performance is controlled and doesn't feel challenged.
	Level 2a	DBL stance	2x5	
	Level 2b	DBL Stance	2x10	
	Level 2c	DBL stance	2x15	
	Level 3a	SL Stance (wall support)	2x5	
	Level 3b	SL Stance (wall support)	2x10	

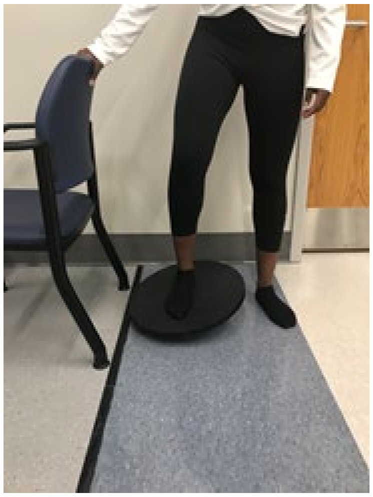

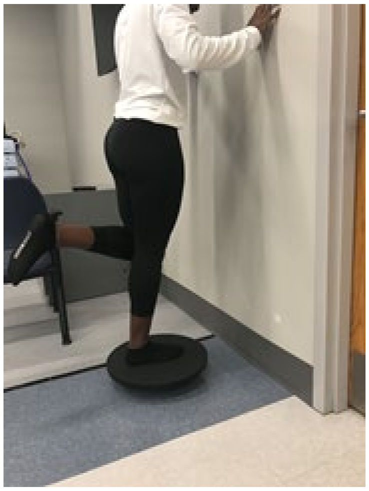

Goal #2: Improve Hip and Ankle Strength

a. **Ankle Isotonics with Resistance Band**. Exercise will be performed in all 4 directions (dorsiflexion, plantarflexion, inversion, eversion). It is preferable to anchor the band around a sturdy chair/table so the patient can practice the same way they will perform the task during home sessions. The patient will be instructed to avoid allowing the band to pull them back to the starting the position ie "you control the motion. Don't allow the band to control it." The band should be stretched to 70% of its resting length and movement through the range of motion should occur at a consistent pace of approximately 3 to 5 seconds per repetition throughout the full range of motion.

Level	Color	Volume	Progress to next level when movement is controlled, and patient is not challenged.
Level 1a	Green	3 x 10	
Level 1b	Green	4 x 10	
Level 2a	Blue	3 x 10	
Level 2b	Blue	4 x 10	
Level 3a	Black	3 x 10	
Level 3b	Black	4 x 10	

Dorsiflexion

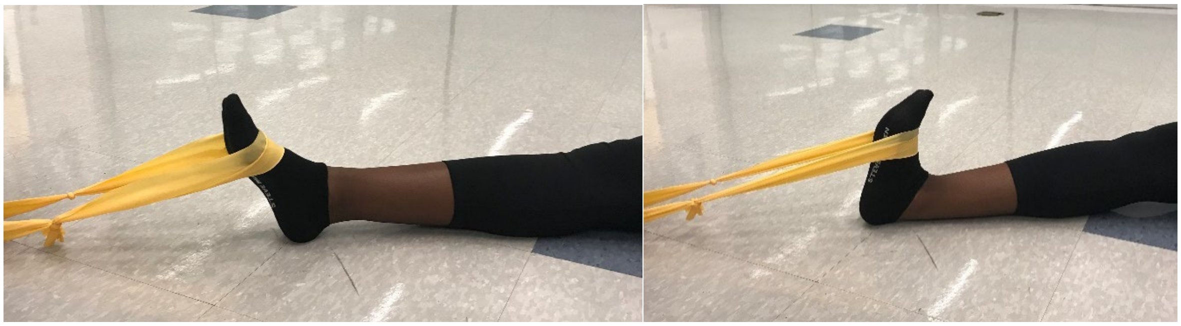

Inversion

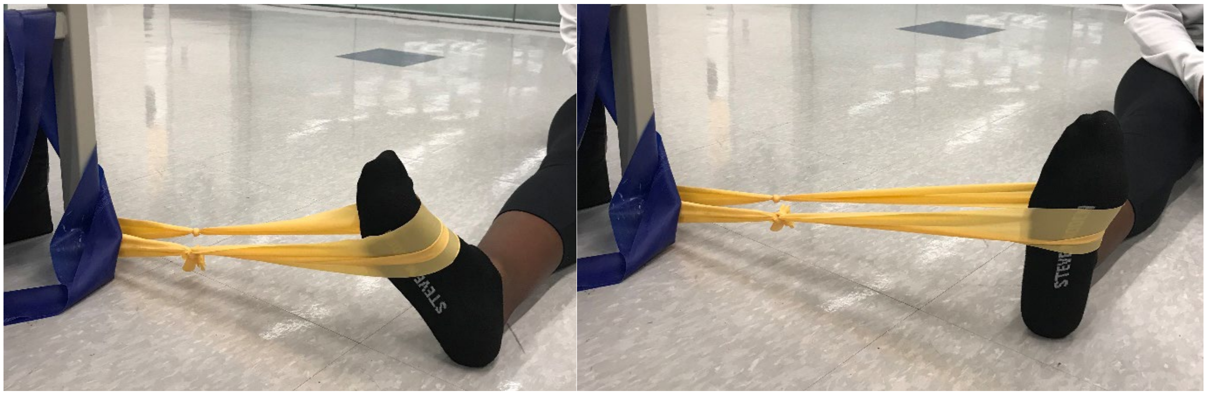

Eversion

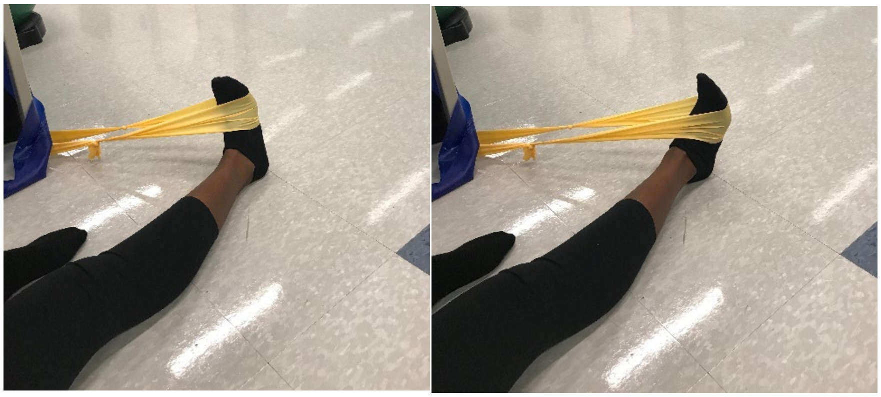

Plantarflexion

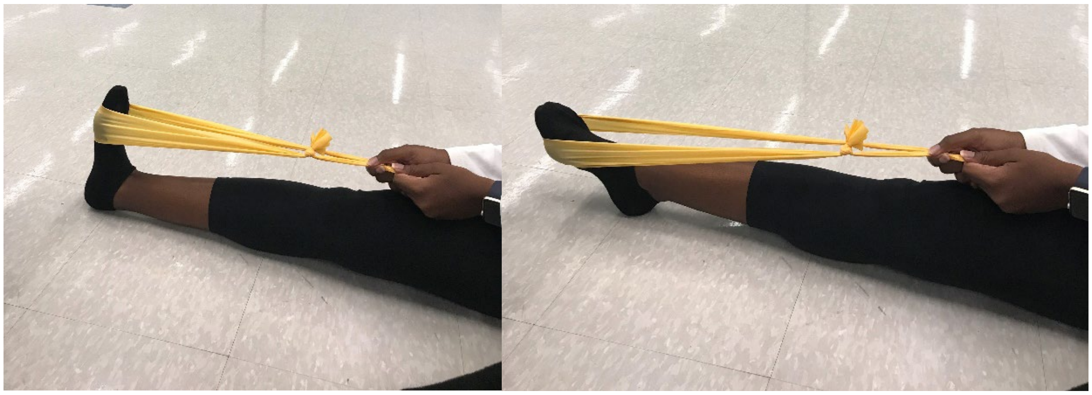

b. Proprioceptive Neuromuscular Facilitation

A slow-reversal PNF technique local to the ankle comprised of concentric contraction of the antagonist muscle followed by a concentric contraction of the agonist muscle will focus on strengthening the ankle musculature in the D1 and D2 patterns. The D1 pattern consists of two phases: dorsiflexion-inversion (up and in) and plantarflexion-eversion (down and out). The D2 pattern also consists of two phases: dorsiflexion-eversion (up and out) and plantarflexion-inversion (down and in). Manual resistance will be applied by the clinician to the distal aspect of foot at the metatarsal heads. Maximal resistance should be applied continually while encouraging movement through the entire ROM. Each repetition (completion of both phases) should take approximately 3-5 seconds to complete. The lower leg should be stabilized at the knee to prevent hip and knee movement.

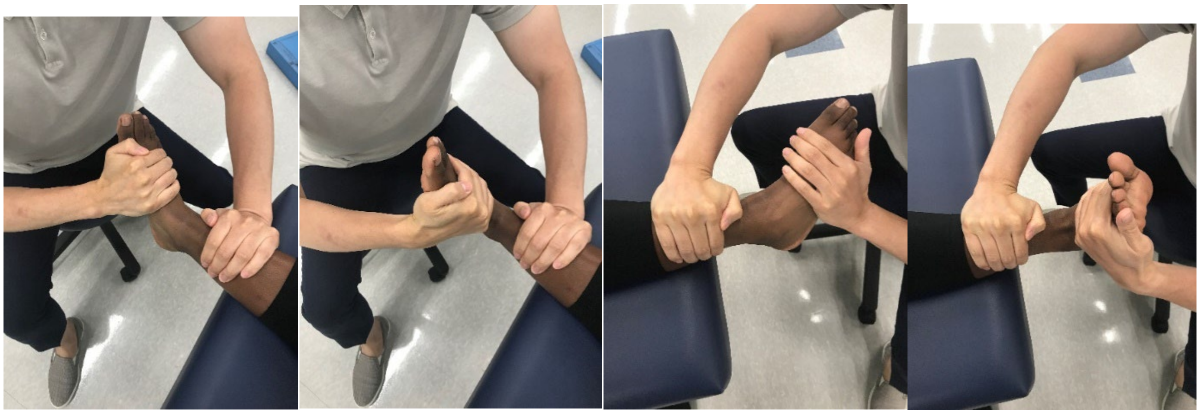

Week	Position	Volume (ea pattern)	Progression will be weekly increases in volume.
1	D1/D2	2 x 10	
2	D1/D2	2 x 15	
3	D1/D2	3 x 10	
4	D1/D2	3 x 15	
5	D1/D2	4 x 10	
6	D1/D2	4 x 15	

c. **Single-Leg Heel Raises**
While standing near a chair or wall for assistance with balance as necessary, the patient will stand on the injured limb and lift the heel up to maximum pain-free plantarflexion, and then lower in the same slow and controlled manner. This task will be progressed to single-leg heel raises off a step with patient's forefoot on an elevated platform or stair to allow for increased range of motion. Patient is instructed to emphasize a slow and controlled lowering/eccentric phase.

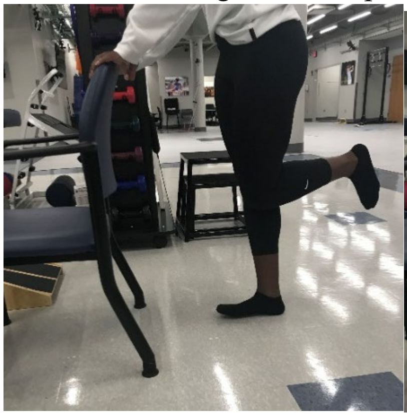
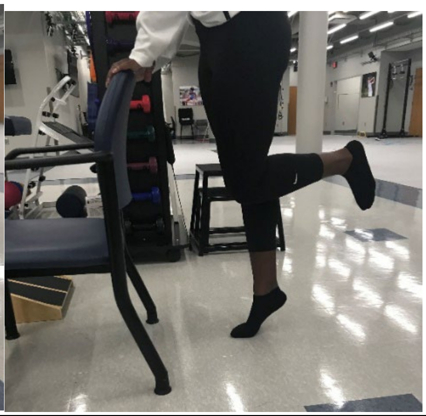

Level	Position	Volume	Progress to next level when movement is controlled, and pt is not challenged.
Level 1a	Floor	3 x 10	
Level 1b	Floor	3 x 15	
Level 2a	Box	2 x 10	
Level 2b	Box	2 x 15	
Level 2c	Box	2 x 20	

d. **Single-leg Kicks (Steamboats)**
While standing on the involved limb and a light resistance band anchored low and placed around the distal leg, the patient moves against the band and returns to start. The motion is 1-second count, controlled return (eccentric) and the patient avoids holding onto anything for balance.

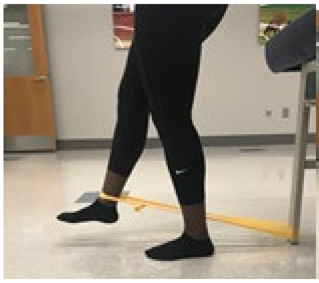
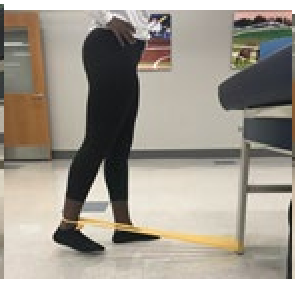
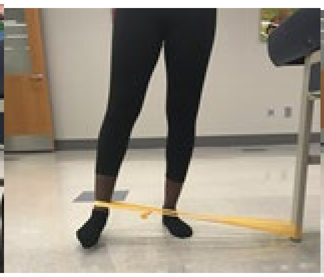
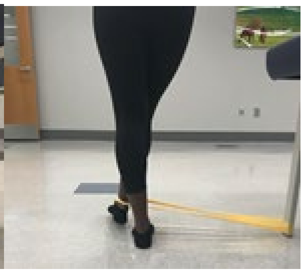

Level	Color	Volume	Progress to next level when movement is controlled, and patient is not challenged.
Level 1a	Green	3 x 10	
Level 1b	Green + Foam Pad	3 x 10	
Level 2a	Blue	3 x 10	
Level 2b	Blue + Foam Pad	3 x 10	
Level 3a	Black	3 x 10	
Level 3b	Black + Foam Pad	3 x 10	

e. Rotational Lunge

Set Up: Patient sits on floor with legs extended heels touching a wall. A mark is place on floor to show the leg-length, which will be the near-foot starting point for the exercise.

Task: Patient will stand with both feet parallel, with the lateral border of the near-foot at the previously placed mark. The patient will be instructed to pick up that foot, externally rotate 90 degrees and lunge with the toes and knee pointing towards the wall (perpendicular to starting position). Where the patient is able to touch the wall with the knee (60-90 degrees of knee flexion), an additional mark is placed on the wall to give the patient a point of reference for consistency. The end of the repetition is the return to the starting position. The patient is instructed to consider a 4-count execution to aid in consistent and controlled movement:

1. Move from starting position to foot placement
2. Lunge forward (knee flexes and touches wall)
3. Knee extends and begin return to start
4. Return to starting position

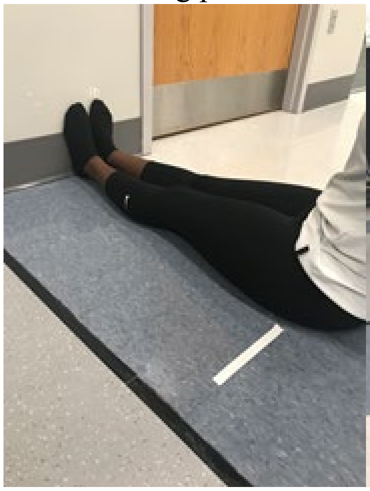
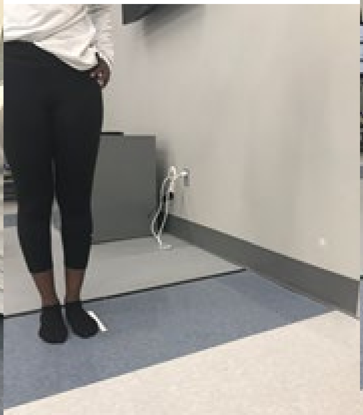
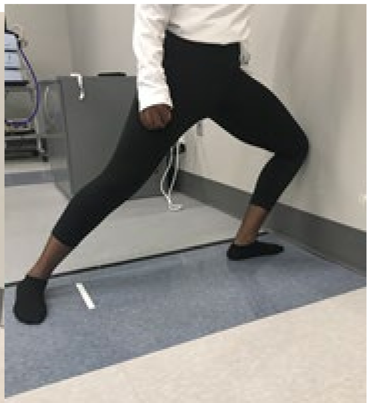

Level	Position	Volume	Progress to next level when movement is controlled, and pt is not challenged.
Level 1a	Floor	2 x 10	
Level 1b	Floor	2 x 15	
Level 1c	Floor	2 x 20	
Level 2a	Foam Pad (at wall)	2 x 10	
Level 2b	Foam Pad (at wall)	2 x 15	
Level 2c	Foam Pad (at wall)	2 x 20	

f. Rotational Squat

Set Up: The patient stands arms distance (tip of 3rd digit in contact with the wall) and a mark is place on the wall at the height of the patient's lateral femoral condyle.

Task: Patient will stand on the near-limb, squat down and rotate laterally, placing stance hip in internal rotation, and reach towards the target with both hands (but is permitted to concentrate on the near hand). The end of the repetition is the return to the starting position, remaining on one limb for the entire exercise. They may place to other foot down in between repetitions. The patient is instructed to consider a 4-count execution to aid in consistent and controlled movement:

1. Move from starting position to single leg squat
2. Rotate to wall and tap target
3. Rotate back to neutral hips, single leg-squat
4. Return to starting position

This exercise is performed bilaterally.

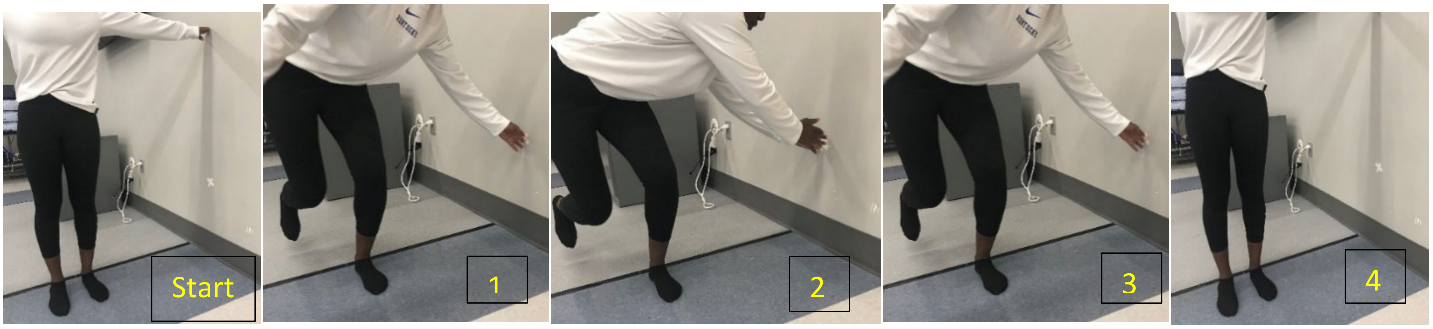

Level	Position	Volume	Progress to next level when movement is controlled, and pt is not challenged.
Level 1a	Floor	2 x 10	
Level 1b	Floor	2 x 15	
Level 1c	Floor	2 x 20	
Level 2a	Foam Pad (at wall)	2 x 10	
Level 2b	Foam Pad (at wall)	2 x 15	
Level 2c	Foam Pad (at wall)	2 x 20	

Goal #3: Improve Static Balance

a. **Single Limb Balance Progression - Eyes Open**

Patients will complete a single limb balance progression which incorporates changes in visual status, stance surface, and stance duration. However, to be considered for progression, the patient must not exhibit any errors (rather than progressing off of time alone.)

Errors are the same as for the Balance Error Scoring System: taking one or both hands off the hips, touching down with the opposite limb, trunk flexion or lean greater than 30 degrees.

For progression on stable surfaces, the patient must not exhibit any errors.

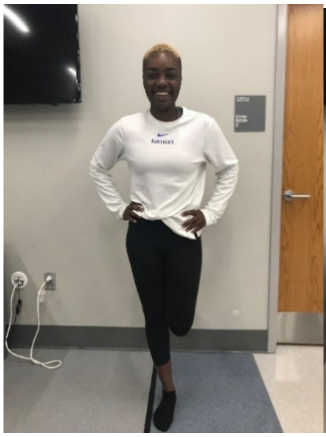
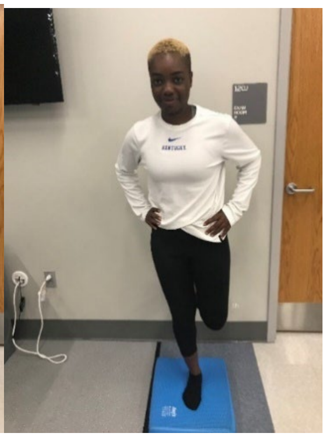

Eyes Open			
Level	Position	Volume	Progression when no errors (see above)
Level 1	Floor	3 x 60s	
Level 2a	Foam Pad	3 x 30s	
Level 2b	Foam Pad	3 x 60s	
Level 2c	Foam Pad	3 x 90s	

b. **Single Limb Balance Progression - Eyes Closed**
Patients will complete a single limb balance progression which incorporates changes in stance surface, and stance duration. However, to be considered for progression, the patient must not exhibit any errors (rather than progressing off of time alone.)

Eyes Closed			
Level	Position	Volume	Progression when no errors (see description above)
Level 1a	Floor - arms out	3 x 30s	
Level 1b	Floor – arms across chest	3 x 30s	
Level 1c	Floor – arms across chest	3 x 60s	
Level 2a	Foam pad – arms out	3 x 30s	
Level 2b	Foam pad – arms across chest	3 x 30s	
Level 2c	Foam pad – arms across chest	3 x 60s	
Level 2d	Foam pad – arms across chest	3 x 90s	

Goal #4: Improve Dynamic Balance

a) Hop to Stabilization

The patient hops 18 inches anteromedial, stabilizes briefly, and then hops back to starting position. The movement is repeated to the anterolateral direction and back. The patient should be positioned forward through each trial. Progressions will be made to the next hop distance when the participant can complete the hop series error-free with their hands on hips.

Anterolateral:

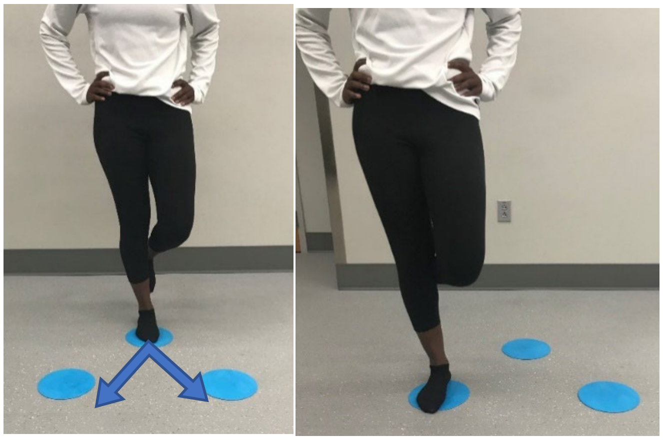

Anteromedial:

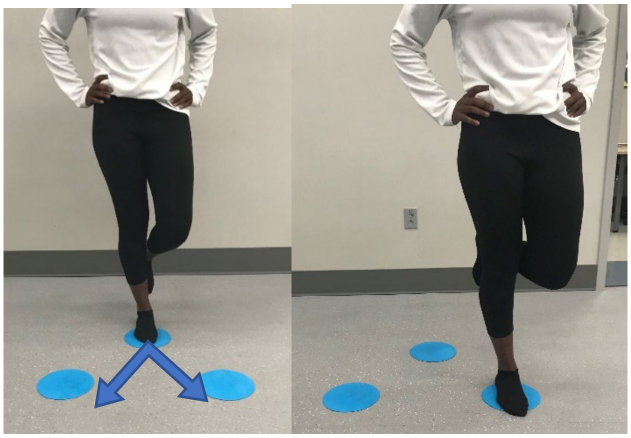

Anterior-Posterior:

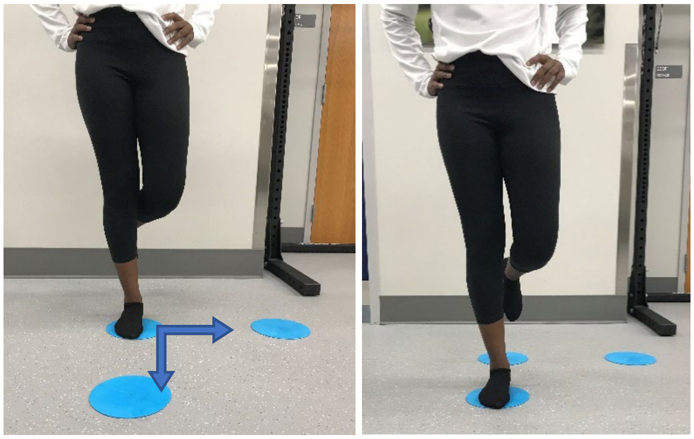

Medial-Lateral:

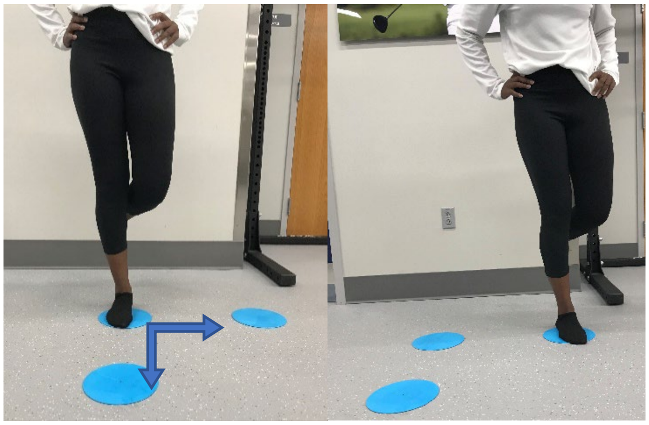

Level	Position	Volume (per direction)	Progress to next level when error-free in each direction
Level 1	18 in – Arms out	1 x 10	
Level 2	18 in – Hands on hips	1 x 10	
Level 3	27 in – Arms out	1 x10	
Level 4	27 in – Hands on hips	1 x 10	
Level 5	36 in – Arms out	1 x10	
Level 6	36 in – Hands on hip	1 x 10	
Level 7	36 in – from 6in platform	1 x 10	

b) Hop to Stabilization and Reach

This task is similar to the Hop to Stabilization task but after stabilization in the single-limb stance, participants had to reach back to the starting position, hop back to the starting position, and then reach to the target position. Participants were not able to advance to the next level in each direction until they demonstrated five repetitions error-free while maintaining hands on hips.

Anterolateral:

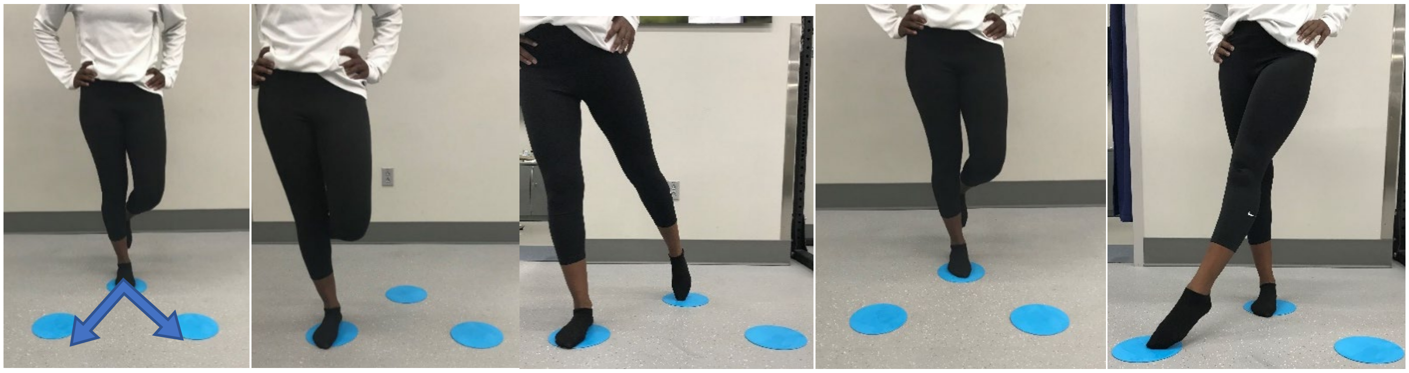

Anteromedial:

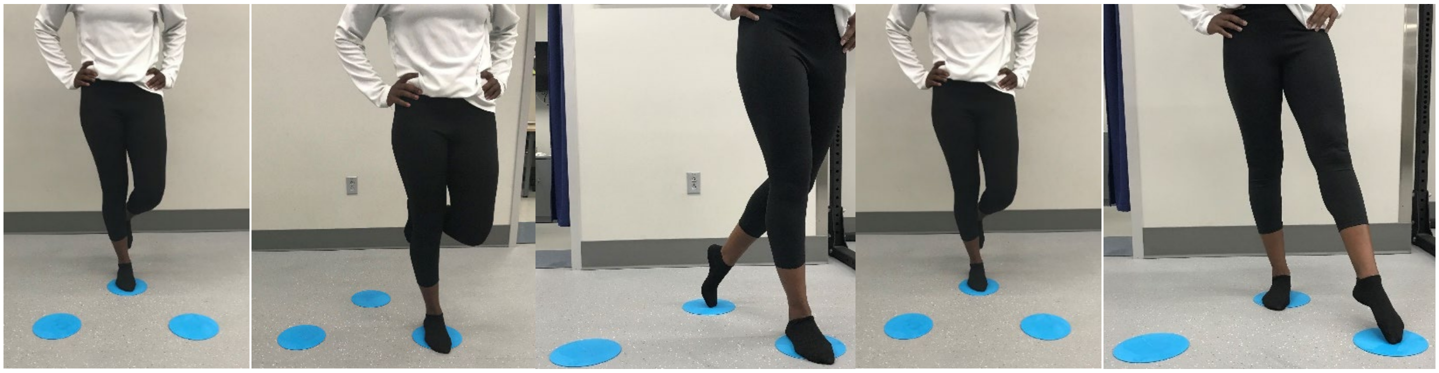

Anterior-Posterior:

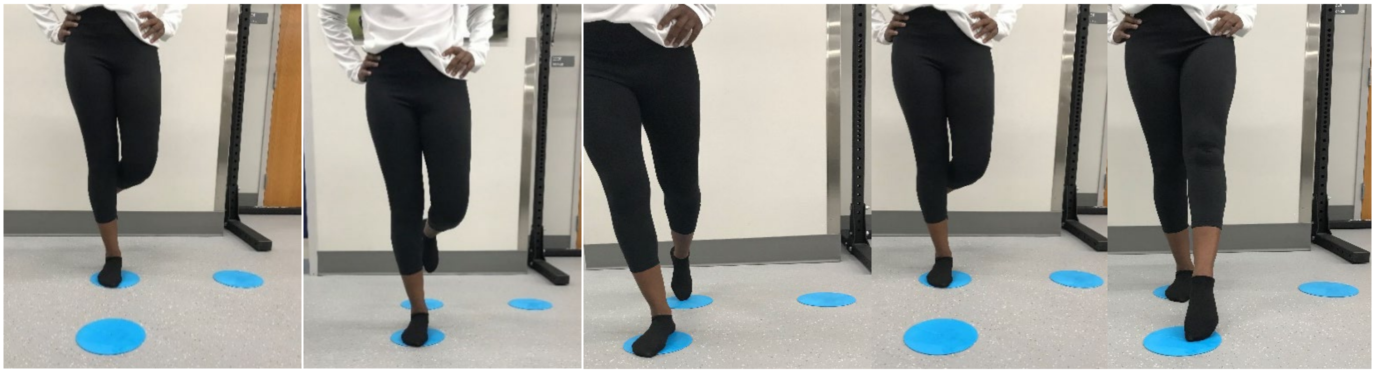

Medial – Lateral:

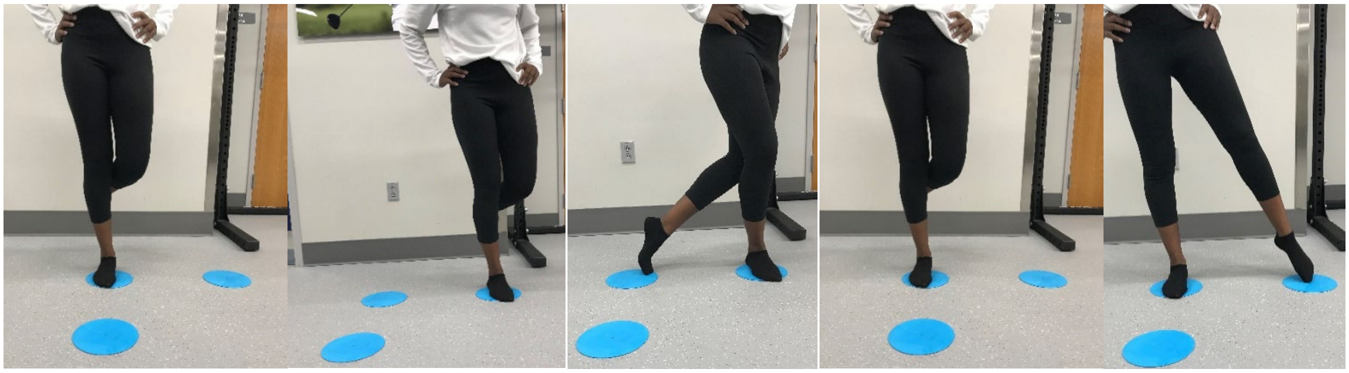

Level	Position	Volume (per direction)	Progress to next level when error-free in each direction
Level 1	18 in – Arms out	1 x 10	
Level 2	18 in – Hands on hips	1 x 10	
Level 3	27 in – Arms out	1 x 10	
Level 4	27 in – Hands on hips	1 x 10	
Level 5	36 in – Arms out	1 x 10	
Level 6	36 in – Hands on hip	1 x 10	
Level 7	36 in – from 6in platform	1 x 10	

c) **Unanticipated Hop to Stabilization**
With a grid on the floor (numbered rubber discs are ideal for this) of 9 markers placed 18 inches apart, the clinician will call out numbers in a random order, but adjacent to the number the patient is standing on. The goal is 3 sets of 10 hops allowing 5 seconds to complete each hop, progressing to a 3 second time limit, and eventually a 1 second time limit. Additional progressions will be introduced by integrating a foam pad(s) to one or more numbers.

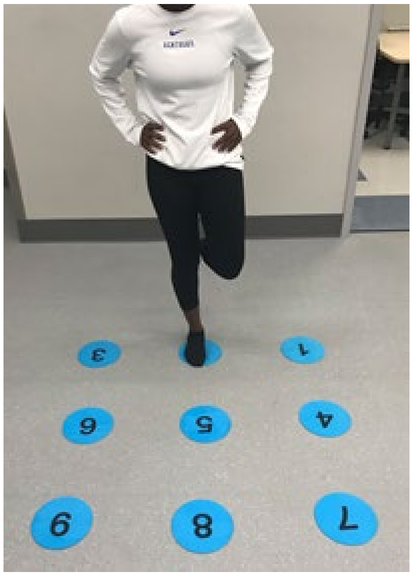

The errors for all hop tasks included:

- Touching down with opposite limb
- Excessive trunk motion (30°- lateral flexion)
- Removal of hands from hips during hands on hips activities
- Bracing the nonstance limb against the stance limb
- Missing the target

The premise for these tasks is the same as described in SOC protocol. However, for progression, the patient must not exhibit any errors and <u>maintain intrinsic foot muscle activation following stabilization</u>. This will be observed by examining motion of the navicular tuberosity and medial longitudinal arch contact with the ground.

Errors and progression criteria should be followed based on the appendix material in McKeon 2008 Med Sci Sport Exer			
Level	Position	Volume	Progress to next level when error-free; progress each direction individually
Level 1	5 seconds (a)	3 x 10	
Level 2	3 seconds (b)	3 x 10	
Level 3	1 second (c)	3 x 10	
Level 4a-c	Add foam pad to one number	3x10	

Standard of Care Home Exercise Library

This library is your reference for the exercises that may be included in your home exercise plan. You may or may not be prescribed all of these exercises for each session. Please refer to your Home Exercise Prescription or contact your provider with questions.

Calf Stretching Option 1 (front leg is stretching)

Stand near a wall with the forefoot of you injured foot against the wall. Lean forward with your knee straight until you feel a gentle stretch in our calf muscle and calf. Return to the starting position and repeat with the front knee bent.

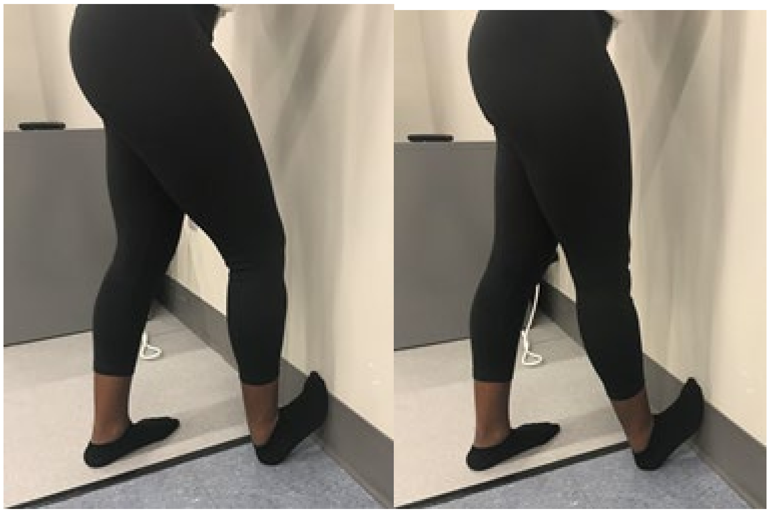

Calf Stretching Option 2 (Back leg is stretching)

Stand with feet shoulder width apart and your non-injured foot forward, with both feet pointing straight ahead. Keep back leg straight and lean forward, bending the knee of your front leg. Make sure you keep your heels flat on the floor. Drift forward until you feel a pull behind your knee or calf and hold. Return to starting position, and repeat with the back leg bent.

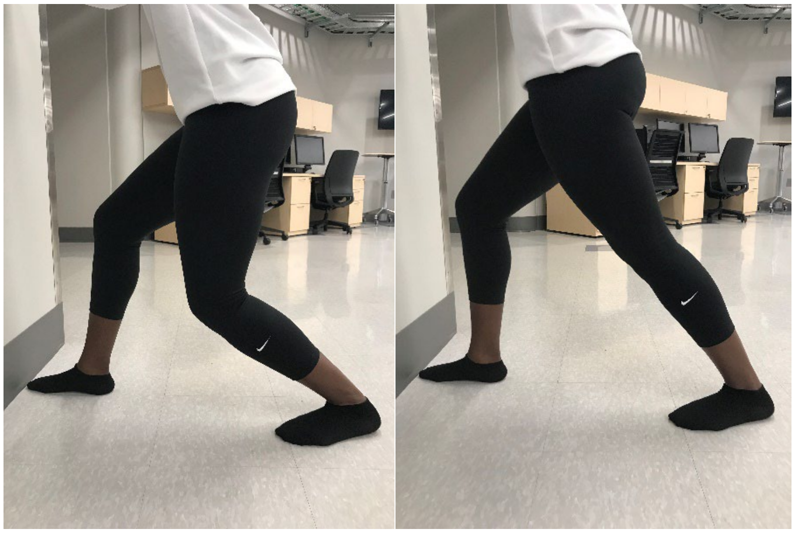

Ankle 4-Way with Band

All theraband exercises are slow and controlled. Do not let the band "bounce" back. Anchor your band around a sturdy table or bed post, or by closing a door with the knot on the other side. When you move to the end of the motion, hold the contraction for 3 seconds. Additionally, try to replicate the resistance of the band that you are experiencing during supervised rehabilitation sessions.

A. Plantarflexion: "gas pedal." Keep knee straight. Place the loop of the band around "ball of foot" and press it away as far as possible, hold for 3 sec, and slowly return to neutral. Repeat.

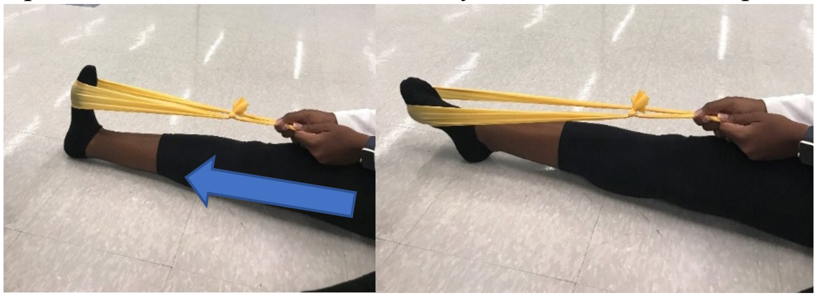

B. Dorsiflexion: start in neutral and pull theraband back toward you as far as possible. Pause for 3 sec. Return slowly. Keep knee straight.

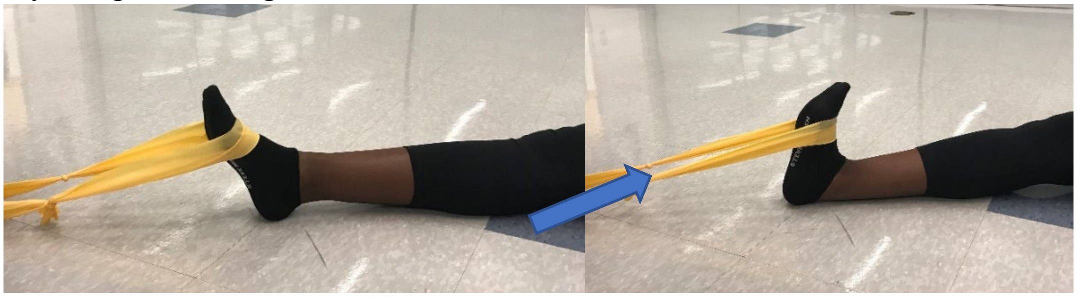

C. Inversion: start neutral and bring band toward your midline without bending or twisting knee. Pause for 3 sec. Return slowly.

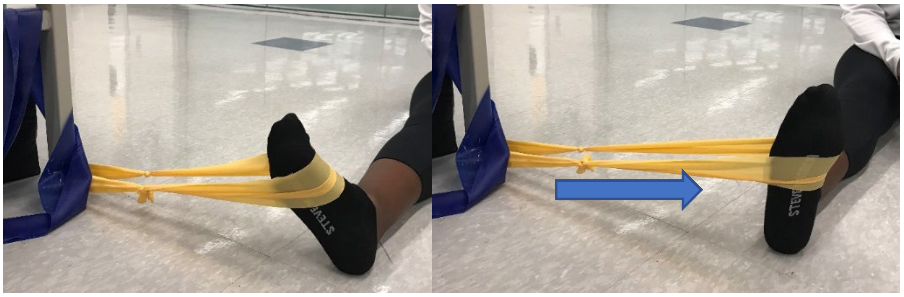

D. Eversion: start neutral and press band out without bending or twisting knee. Pause for 3 sec. Return slowly.

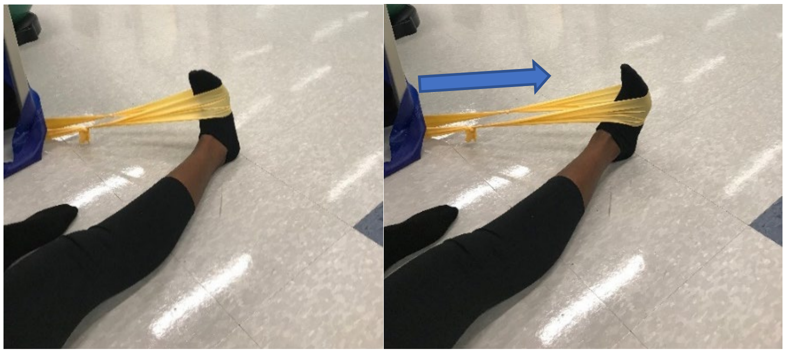

Single Leg Balance

Practice balancing on your non-injured leg (barefoot), keeping your hands on your hips. Once you can balance for 20 seconds with your eyes open, practice with eyes closed. Tip: find your balance first with your eyes open.

Then practice on a non-stable surface using the balance pad provided.

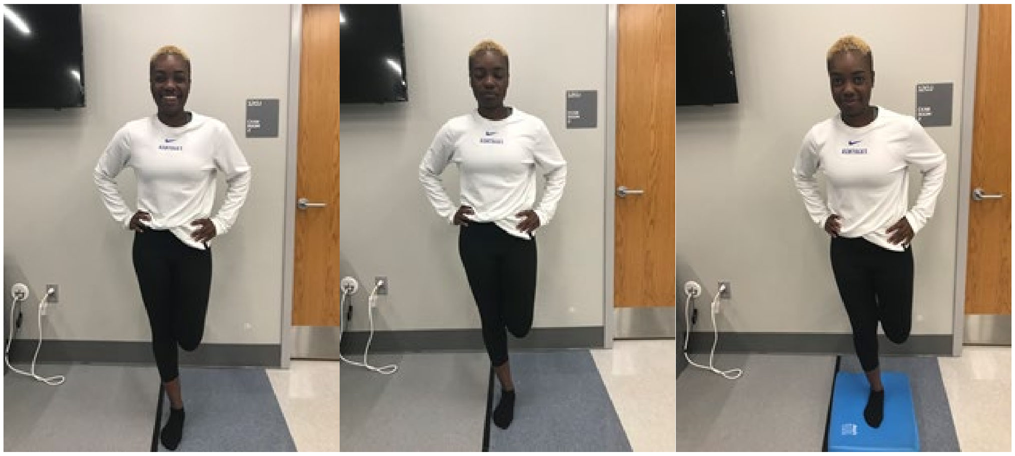

Side-Lying Glute Strengthening
While lying on your side, slowly raise up your top leg to the side. Keep your knee straight and your toes pulled up towards your shin the entre time. Keep your leg in line with your body and your hips stacked; do not let your torso roll forward or back. The bottom leg should be bent to stabilize your body. Hold this position for 3 sec and then slowly lower your top leg. This exercise will be progressed by adding a resistance band around both thighs.

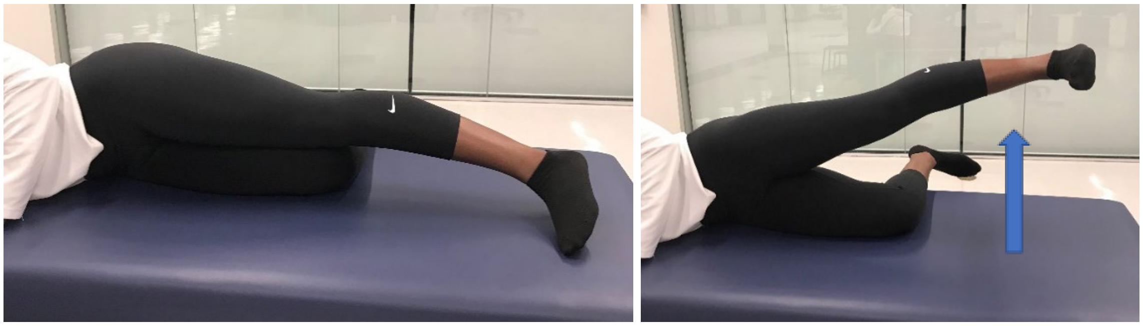

Prone Leg Extension
While lying face down with your knee bent, slowly raise your knee off the ground. Try to keep both front hip bones in contact with the ground and avoid twisting at your waist.

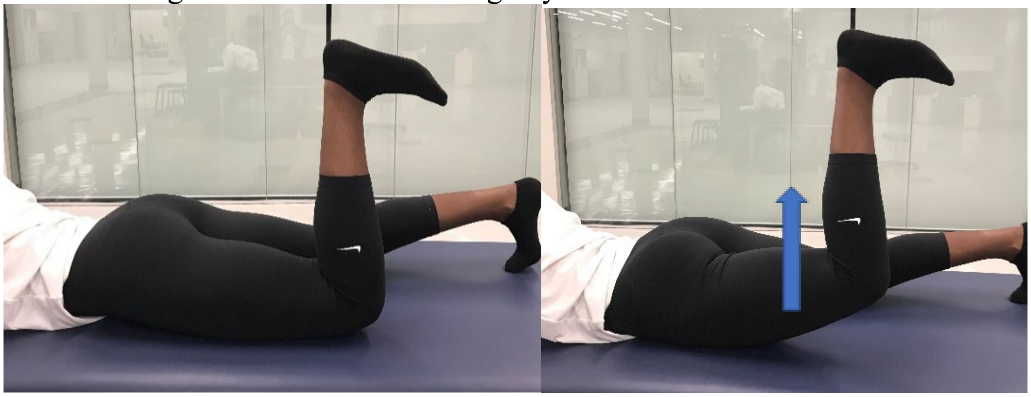

Rotational Squat
Place a post-it note or piece of tape on the wall at the level of the bony prominence below your knee cap (tibial tuberosity). Stand one arm's length away from the wall with the injured leg side closest to the wall. This move is four-count. 1) On the leg closest to the wall, squat down while keeping your hips facing forward. 2) Rotate towards the wall and tap your mark with the closest hand. If you cannot reach it, you most likely need to squat lower next time. 3) Return to facing forward. 4) Straighten your knee to return to standing.

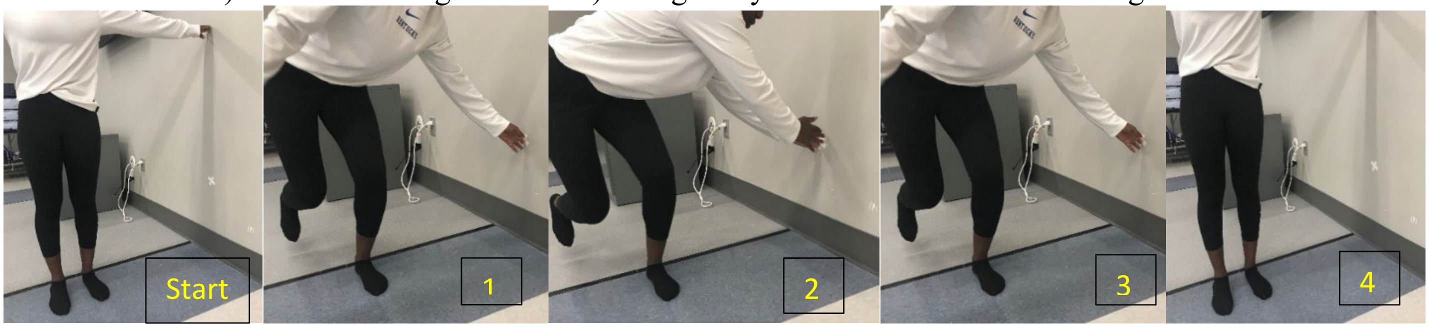

Rotational Lunge
Using the same mark on the wall as the rotational squat, start by standing parallel to the wall one leg's distance from the wall. Stand so that your injured side is closest to the wall. Step towards the wall, and bend that leg to tap your mark on the wall with the front of your knee. Return to staring position.

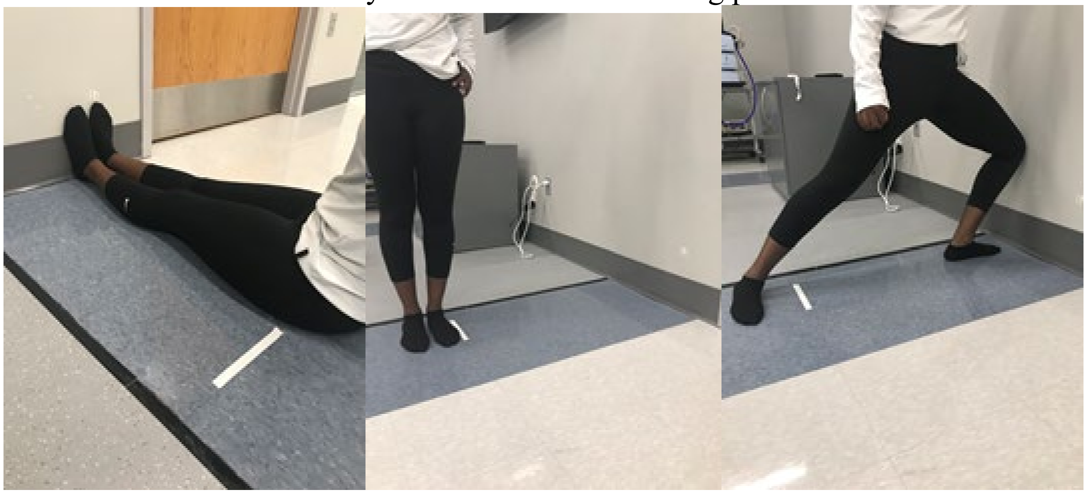

Supplement: Supplementary file 1 — Additional file 1. Rehabilitation guide for the supervised and home exercises utilized for the Foot Intensive Rehabilitation (FIRE) and Standard of Care (SOC) groups. [file 13102_2023_667_MOESM1_ESM.pdf]
